# Identification of the allosteric P2X_7_ receptor antagonist [^11^C]SMW139 as a PET tracer of microglial activation

**DOI:** 10.1038/s41598-018-24814-0

**Published:** 2018-04-26

**Authors:** Bieneke Janssen, Danielle J. Vugts, Shane M. Wilkinson, Dieter Ory, Sylvie Chalon, Jeroen J. M. Hoozemans, Robert C. Schuit, Wissam Beaino, Esther J. M. Kooijman, Johan van den Hoek, Mansoor Chishty, Aurélie Doméné, Anke Van der Perren, Alessandro Villa, Adriana Maggi, Ger T. Molenaar, Uta Funke, Rostislav V. Shevchenko, Veerle Baekelandt, Guy Bormans, Adriaan A. Lammertsma, Michael Kassiou, Albert D. Windhorst

**Affiliations:** 1 Department of Radiology & Nuclear Medicine, Neuroscience Campus Amsterdam, VU University Medical Center, Amsterdam, The Netherlands; 2 School of Chemistry, University of Sydney, Sydney, Australia; 3 Laboratory for Radiopharmaceutical Research, Department of Pharmaceutical and Pharmacological Sciences, KU Leuven, Leuven, Belgium; 4 UMR 1253, iBrain, Université de Tours, Inserm, Tours, France; 5 Department of Pathology, VU University Medical Center, Amsterdam, The Netherlands; 6 Pharmidex Pharmaceutical Services Ltd., London, United Kingdom; 7 Neurobiology and Gene Therapy, Department of Neurosciences, KU Leuven, Leuven, Belgium; 8 Center of Excellence on Neurodegenerative Diseases and Department of Pharmacological and Biomolecular Sciences, University of Milan, Milan, Italy; 9 BV Cyclotron VU, Amsterdam, The Netherlands

## Abstract

The P2X_7_ receptor plays a significant role in microglial activation, and as a potential drug target, the P2X_7_ receptor is also an interesting target in positron emission tomography. The current study aimed at the development and evaluation of a potent tracer targeting the P2X_7_ receptor, to which end four adamantanyl benzamide analogues with high affinity for the human P2X_7_ receptor were labelled with carbon-11. All four analogues could be obtained in excellent radiochemical yield and high radiochemical purity and molar activity, and all analogues entered the rat brain. [^11^C]SMW139 showed the highest metabolic stability in rat plasma, and showed high binding to the *h*P2X_7_ receptor *in vivo* in a *h*P2X_7_ receptor overexpressing rat model. Although no significant difference in binding of [^11^C]SMW139 was observed between post mortem brain tissue of Alzheimer’s disease patients and that of healthy controls in *in vitro* autoradiography experiments, [^11^C]SMW139 could be a promising tracer for P2X_7_ receptor imaging using positron emission tomography, due to high receptor binding *in vivo* in the *h*P2X_7_ receptor overexpressing rat model. However, further investigation of both P2X_7_ receptor expression and binding of [^11^C]SMW139 in other neurological diseases involving microglial activation is warranted.

## Introduction

Neuroinflammation plays a central role in a variety of pathologies affecting the central nervous system (CNS), such as neurodegenerative and neuropsychiatric disorders. CNS-specific immune cells, microglia and astrocytes, are the main cellular effectors of neuroinflammation, although peripheral immune cells (monocytes, macrophages and T cells) can also be recruited to a site of insult. Purinergic signalling through P2X and P2Y receptors is involved in regulation and mediation of the neuroinflammatory response. For P2X receptors, the strongest body of evidence for involvement in mediating neuroinflammation exists for the P2X_7_ subtype^1^. Microglia undergo a morphological change upon pathological disturbances in the brain, and the morphological change goes along with a disease-specific phenotype shift, resulting in alteration of expression of e.g. cell surface receptors, one of which is the P2X_7_ receptor (P2X_7_R)^2^. Although P2X_7_R expression has also been demonstrated on neurons, oligodendrocytes and astrocytes, the highest levels of expression are found on microglia^2,3^. The affinity of its natural ligand adenosine triphosphate (ATP) for P2X_7_R is low and therefore the receptor is insensitive to micromolar changes in extracellular ATP concentrations. On the other hand, millimolar changes in ATP concentrations, e.g. in the case of cell damage or death, leads to activation of the receptor. P2X_7_R signalling is involved in the activation of the inflammasome and the release of the inflammatory cytokine interleukin 1β. Prolonged stimulation of P2X_7_R by ATP results in over-activation of the receptor, leading to pore formation, which allows for transport of macromolecules of up to 900 Da.^1,3^. Due to its up-regulation in CNS pathologies such as Alzheimer’s disease (AD) and multiple sclerosis (MS), P2X_7_R is considered to be a promising drug target for blockade of the neuroinflammatory cascade^2^. In line with this, P2X_7_R is also emerging as a target in positron emission tomography (PET), with four radiolabelled antagonists reported to date^4–9^. Recently, Wilkinson *et al*. reported a series of benzamides as potent P2X_7_R antagonists^10^, based on previously developed benzamide **1**^11^. In this study four of these benzamide analogues (**1**, **2**, **3** and SMW139, with *K*_i_ values of respectively 9, 22, 23 and 32 nM for human P2X_7_R) were radiolabelled and evaluated *in vivo* in healthy rats. The most promising radiotracer, [^11^C]SMW139, was evaluated in a recently developed rat model overexpressing human P2X_7_R^6^. In addition, to assess the potential of [^11^C]SMW139 in a clinical setting, *in vitro* autoradiography studies on post mortem brain material of AD patients were performed, including extensive immunohistochemical staining.

## Results

### Chemistry

**Figure 1:**
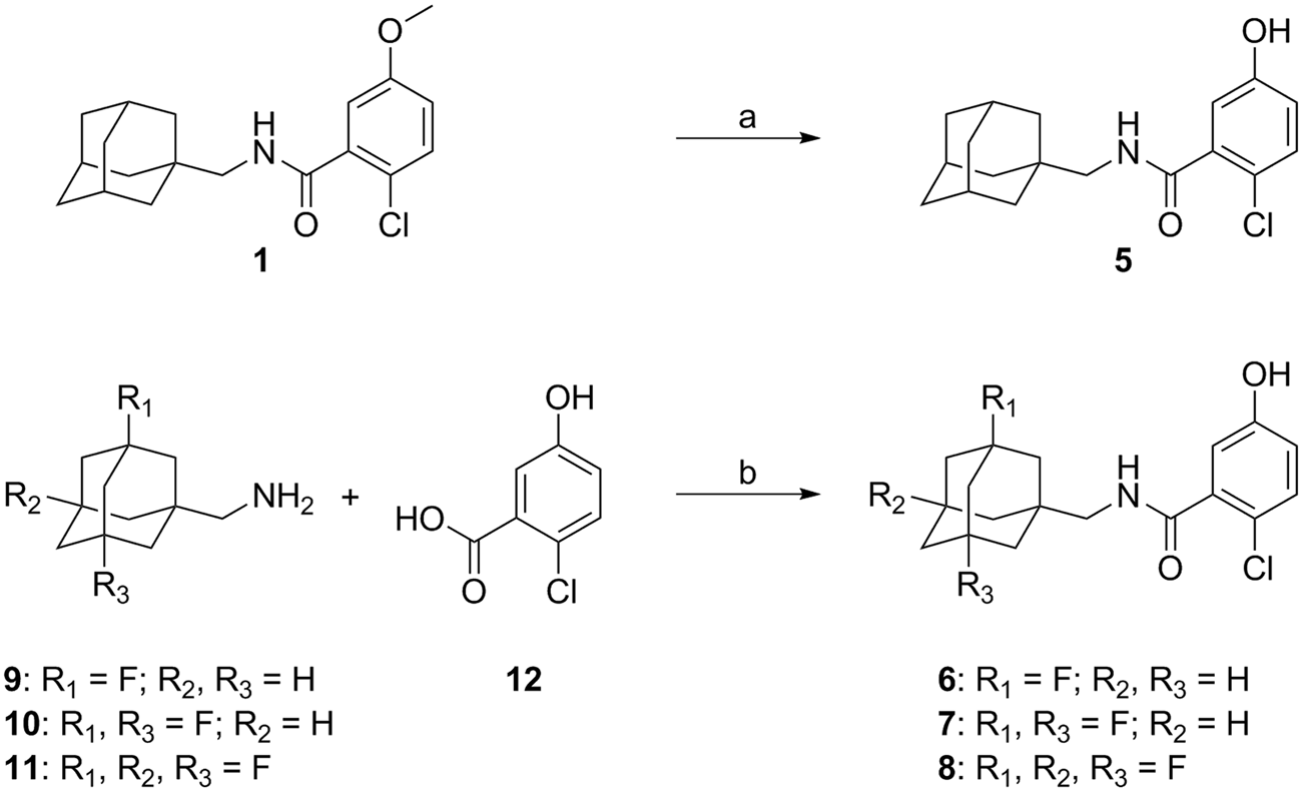
Synthesis of adamantanyl benzamide precursors **5**–**8**. Reagents and conditions: (a) BBr_3_, CH_2_Cl_2_, 0 °C to rt, 48 h, quant. (b) PyBOP, DiPEA, CH_2_Cl_2_, rt, 18 h, 73% (**6**), 77% (**7**), 79% (**8**).

Benzamide precursor **5** was obtained via demethylation of benzamide **1** (Fig. 1) in quantitative yield and with high purity. As for the fluorinated adamantanyl analogues, demethylation using boron tribromide would lead to trans-halogenation of the fluorine, analogues **6**–**8** were synthesised in good yields (73–79%) via a chemoselective coupling of 2-chloro-5-hydroxybenzoic acid **12** and the appropriate adamantanemethylamine (**9**–**11**, Fig. 1).

### Radiochemistry

**Figure 2:**
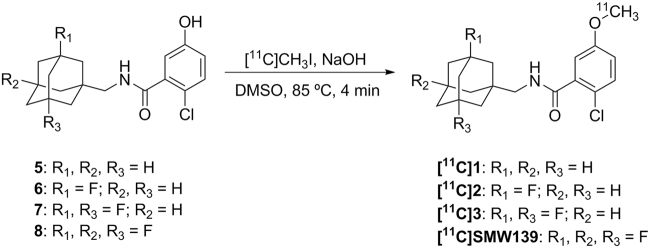
Carbon-11 methylation of benzamide analogues **1**–**3** and [^11^C]SMW139.

Desmethyl benzamide analogues **5–8** were subjected to carbon-11 methylation (Fig. 2) by reaction with [^11^C]methyl iodide in the presence of sodium hydroxide at elevated temperature. After purification by high performance liquid chromatography (HPLC), [^11^C]**1–3** and [^11^C]SMW139 were all obtained in good decay corrected (d.c.) radiochemical yields (RCY) and radiochemical purity (RCP) and high molar activity (A_m_), listed in Table 1. For [^11^C]**1**, the collected fraction (eluent: 50 mM NH_4_OAc (pH 10)/ethanol (EtOH)) was neutralised with 1 mL of citrate buffer and then further diluted with 7.09 mM NaH_2_PO_4_ in saline to contain ≤10% EtOH before use *in vivo*. For [^11^C]**2**, **3** and [^11^C]SMW139, the collected fraction was subjected to solid phase extraction before use *in vivo*, resulting in a formulation solution of the products in ≤10% EtOH in 7.09 mM NaH_2_PO_4_. Total synthesis time for all tracers was 35–40 minutes. All product formulations were stable up to 2 hours, as determined by analytical HPLC.

**Table 1:** Radiolabelling results and LogD_oct,7.4_ of [^11^C]**1**–**3** and [^11^C]SMW139. Values are given as mean ± standard deviation.

Product	Isolated yield (GBq)*	RCY (%, d.c.)	RCP (%)	A_m_ (GBq·µmol^−1^)*	LogD_oct,7.4_ (n = 3)
**[**^**11**^**C]1** (n = 6)	5.0 ± 1.7	33 ± 9	97 ± 1	159 ± 77	2.88 ± 0.14
**[**^**11**^**C]2** (n = 5)	3.9 ± 0.8	30 ± 8	97 ± 1	84 ± 59	2.93 ± 0.02
**[**^**11**^**C]3** (n = 3)	5.1 ± 0.5	41 ± 9	99 ± 0	239 ± 90	2.71 ± 0.01
**[**^**11**^**C]SMW139** (n = 10)	4.8 ± 1.0	37 ± 9	96 ± 2	209 ± 83	2.70 ± 0.01

^*^Measured at end of synthesis.

### Determination of LogD_oct,7.4_

The LogD_oct,7.4_ of analogues [^11^C]**1–3** and [^11^C]SMW139 ([^11^C]**4**) was determined using the shake-flask method (partitioning between 1-octanol and 0.2 M phosphate buffer (pH 7.4)). Determined LogD_oct,7.4_ values (Table 1) show the same trend as LogD values reported by Wilkinson *et al*.^10^.

### *Ex vivo* biodistribution

**Figure 3:**
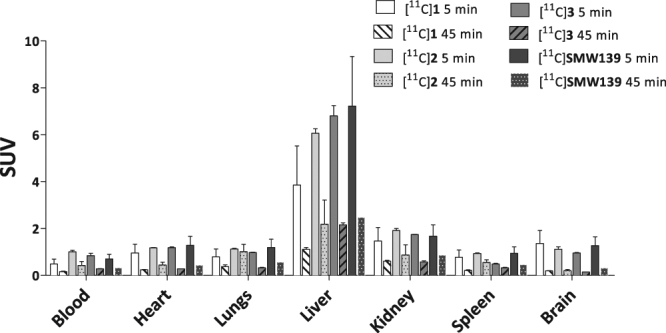
*Ex vivo* biodistribution following intravenous administration of 13–23 MBq of [^11^C]**1–3** and [^11^C]SMW139 in healthy male Wistar rats (n = 3 per tracer per time point). Good initial brain uptake was observed for all four analogues. Data are expressed as standardized uptake value (SUV) ± standard error of the mean (SEM).

All four analogues entered the rat brain and no abnormalities in peripheral uptake were observed (Fig. 3). In striatum, initial standardized uptake values (SUVs) were 1.07–1.46 at 5 min post injection (p.i.) and decreased to 0.14–0.32 at 45 min p.i. All analogues primarily showed clearance via the liver. For clarity, Fig. 3 only shows distribution of [^11^C]**1–3** and [^11^C]SMW139 at 5 and 45 min p.i. Full biodistribution data, including data for separate brain regions, are provided in the Supplementary Information (Figures S1–S4).

### Metabolite analysis

*In vivo* stability of analogues [^11^C]**1**–**3** and [^11^C]SMW139 was assessed in healthy male Wistar rats at 15 and 45 minutes after injection of 30–40 MBq of formulated tracer. In plasma, metabolism of [^11^C]**1–3** showed similar patterns (15–25% of intact tracer at 45 min p.i.), but metabolic stability of [^11^C]SMW139 in plasma was enhanced (42% of intact tracer at 45 min p.i.; Table 2). Results obtained in plasma correspond with previously reported stability results in rat liver microsomes^10^. A higher level of intact tracer was found in brain (Table 3) for [^11^C]**1** and [^11^C]SMW139 compared with [^11^C]**2** and [^11^C]**3**. Despite optimisation of the extraction method, the percentage of total radioactivity that could be extracted from brain homogenates was not optimal for all four compounds (59 ± 6% for [^11^C]**1**; 58 ± 7% for [^11^C]**2**; 61 ± 4% for [^11^C]**3**; 62 ± 3% for [^11^C]SMW139). Therefore, based on its highest stability in plasma *in vivo*, [^11^C]SMW139 was selected for further *in vivo* studies. As imaging studies were performed in female rats, plasma metabolites were also determined in two female rats, and no differences were found between plasma metabolism of [^11^C]SMW139 in male and female rats (Table S1; Supplementary Information).

**Table 2:** Metabolic profiles of [^11^C]**1–3** and [^11^C]SMW139 in rat plasma.

Plasma	[^11^C]1		[^11^C]2		[^11^C]3		[^11^C]**SMW139**	
	15 min	45 min	15 min	45 min	15 min	45 min	15 min	45 min
Intact tracer (%)	37 ± 5	25 ± 1	37 ± 7	15 ± 2	35 ± 1	16 ± 1	60 ± 1	42 ± 2
Non-polar metabolites (%)	53 ± 6	39 ± 1	36 ± 8	12 ± 2	29 ± 1	23 ± 1	11 ± 1	14 ± 1
Polar metabolites (%)	10 ± 1	36 ± 1	27 ± 1	73 ± 3	36 ± 1	61 ± 2	29 ± 2	44 ± 2

**Table 3:** Metabolic profiles of [^11^C]**1–3** and [^11^C]SMW139 in rat brain.

Brain	[^11^C]1		[^11^C]2		[^11^C]3		[^11^C]**SMW139**	
	15 min	45 min	15 min	45 min	15 min	45 min	15 min	45 min
Intact tracer (%)	82 ± 1	76 ± 2	67 ± 5	32 ± 4	62 ± 2	32 ± 2	79 ± 1	66 ± 2
Non-polar metabolites (%)	18 ± 1	24 ± 2	33 ± 5	68 ± 4	38 ± 2	68 ± 2	21 ± 1	34 ± 3

### Autoradiography in a rat model overexpressing *h*P2X_7_R

**Figure 4:**
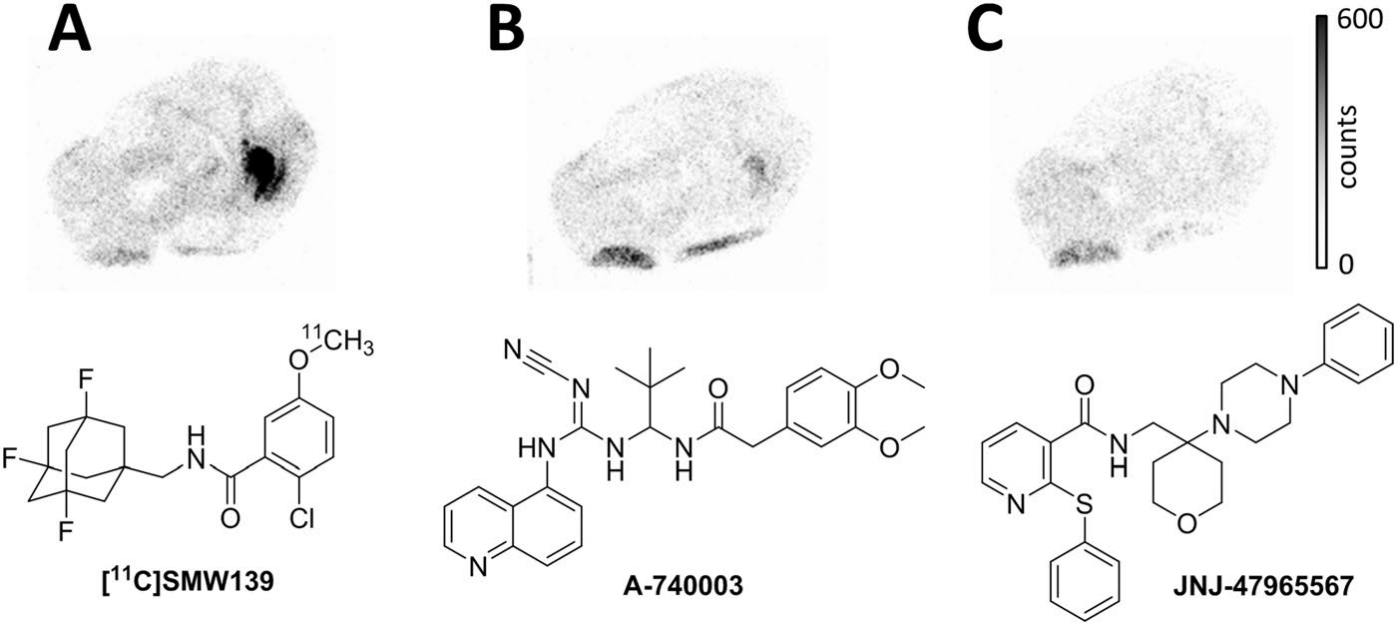
Autoradiograms of transversal cryosections of rAAV-3flag-*h*P2X_7_R vector injected rat brain. (**A**) Increased [^11^C]SMW139 binding is observed in rAAV_3flag-*h*P2X_7_R injected striatum (left on image). (**B**) [^11^C]SMW139 binding in rAAV_3flag-*h*P2X_7_R striatum is blocked (79 ± 10%, n = 2) by co-incubation with A-740003 (10 µM). (**C**) [^11^C]SMW139 binding in rAAV_3flag-*h*P2X_7_R striatum is blocked (93 ± 1%, n = 2) by co-incubation with JNJ-47965567 (10 µM).

**Figure 5:**
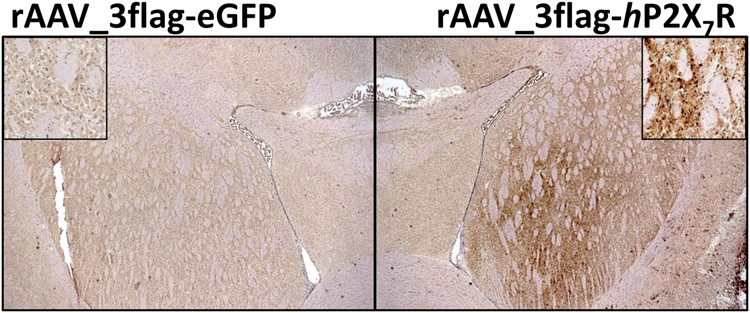
Immunohistochemical staining for P2X_7_R of transversal cryosections of rAAV-3flag-*h*P2X_7_R vector injected rat brain. Left: rAAV_3flag-eGFP injected striatum with magnification in upper left corner. Right: rAAV_3flag-*h*P2X_7_R injected striatum with magnification in upper right corner.

Binding of [^11^C]SMW139 to the human P2X_7_ receptor (*h*P2X_7_R) was assessed *in vitro* on transversal cryosections of rat brains that locally overexpress *h*P2X_7_R, induced by an adeno-associated viral (AAV) vector. Sections incubated with [^11^C]SMW139 (28 nM) showed on average 10-fold increased binding (10 ± 2, n = 2) in the rAAV_3flag-*h*P2X_7_R injected striatum (Fig. 4A; mean intensity 678 ± 55 counts) compared with the rAAV_3flag-eGFP injected striatum (mean intensity 69 ± 10 counts). Co-incubation with two structurally different P2X_7_R antagonists (10 µM; A-740003^12,13^ and JNJ-47965567^14,15^) decreased [^11^C]SMW139 binding in the rAAV_3flag-*h*P2X_7_R injected striatum with 79–93%, depending on the blocker used (Fig. 4B,C). P2X_7_R expression in the rAAV_3flag-*h*P2X_7_R injected striatum was confirmed with immunohistochemical staining (Fig. 5).

### PET imaging

**Figure 6:**
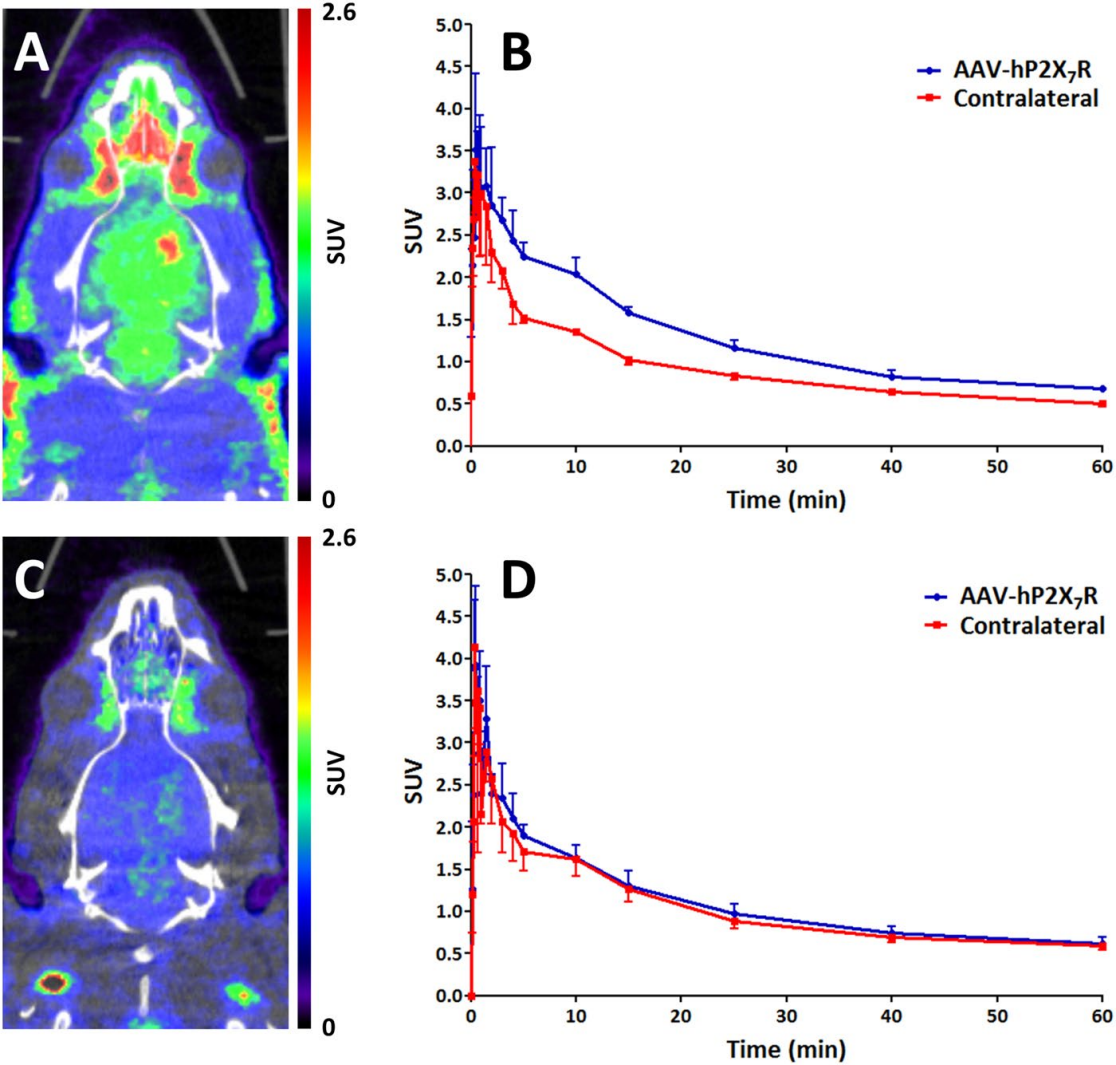
PET imaging with [^11^C]SMW139 in rAAV_3flag-*h*P2X_7_R rat model. (**A**) Representative PET-CT image (summed images 0–60 min) of [^11^C]SMW139 showing increased uptake in rAAV_3flag-*h*P2X_7_R injected striatum compared with rAAV_3flag-eGFP (control vector) injected striatum (n = 3). (**B**) Time-activity curves (TACs) of rAAV_3flag-*h*P2X_7_R injected striatum and contralateral (rAAV_3flag-eGFP injected) striatum. Ellipsoid regions of interest (ROIs) were defined within striatum (dimensions: x = 3.4 mm, y = 3.6 mm, z = 2.9 mm) (**C**) Representative PET-CT image (summed images 0–60 min) of [^11^C]SMW139 after pre-treatment with JNJ-47965567 (30 mg·kg^−1^ s.c., 45 min prior to tracer injection). (**D**) TACs of rAAV_3flag-*h*P2X_7_R injected and contralateral striatum after pre-treatment. Ellipsoid ROIs were defined within striatum (dimensions: x = 3.4 mm, y = 3.6 mm, z = 2.9 mm).

*In vivo* PET imaging was performed in female rats injected with rAAV_3flag-*h*P2X_7_R to induce overexpression of *h*P2X_7_R in the right striatum. Uptake of [^11^C]SMW139 was 1.5-fold higher in the right striatum compared with the contralateral striatum at 4–15 min p.i. (n = 3; Fig. 6A,B, and Supplementary Figure S5, 5 weeks after vector injection). [^11^C]SMW139 uptake in rAAV_3flag-*h*P2X_7_R striatum was significantly increased from 2 min p.i. (2.85 ± 0.69 vs. 2.30 ± 0.36 in contralateral striatum) throughout the remainder of the scanning time. Pre-treatment with JNJ-47965567 (30 mg·kg^−1^)^14,15^ 45 min prior to tracer injection reduced the uptake of [^11^C]SMW139 down to baseline levels (2.40 ± 0.22 at 2 min p.i.; n = 3; Fig. 6C,D, 11 weeks after vector injection).

### Extended single microdose toxicity study

Following intravenous administration of a single dose of non-radiolabelled SMW139 (0.12 mg·kg^−1^), no abnormalities, complications or toxic clinical effects were observed in healthy male and female rats. In both male and female animals, body weight curves were positive and did not differ between vehicle injected animals and animals injected with SMW139 (p ≥ 0.05). In addition, food consumption was stable in all groups. No treatment-related changes were found in the groups receiving SMW139 in any of the parameters used for haematology, coagulation and clinical chemistry (summary of full report in Supplementary Information). No significant differences in organ weights were found between groups and no abnormalities suggesting a toxic effect of SMW139 was observed during necropsy.

### Autoradiography and immunohistochemical staining in post mortem human brain tissue

**Figure 7:**
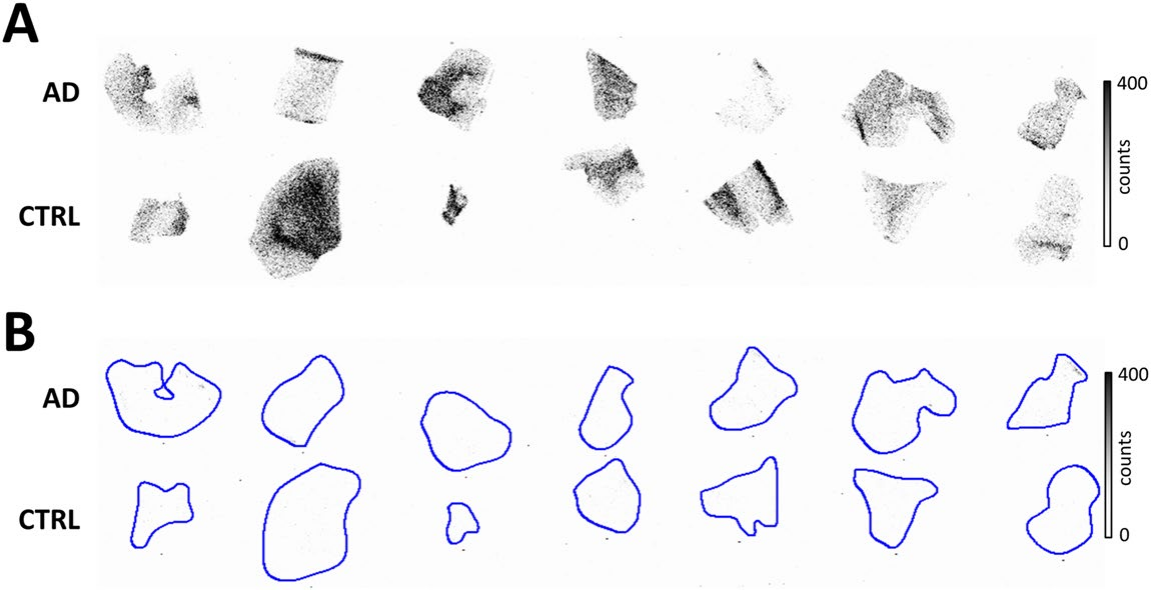
Autoradiograms of post mortem human brain tissue (temporal cortex). (**A**) Total binding of [^11^C]SMW139 (28 nM). Upper row: AD patients, lower row: non-neurological controls (CTRL). Binding of [^11^C]SMW139 does not differ significantly between AD patients and controls. Higher tracer binding was observed in white matter compared with grey matter. (**B**) Binding of [^11^C]SMW139 could be fully blocked with 10 µM of JNJ-47965567 in both AD patients (upper row) and non-neurological age-matched controls (lower row).

**Figure 8:**
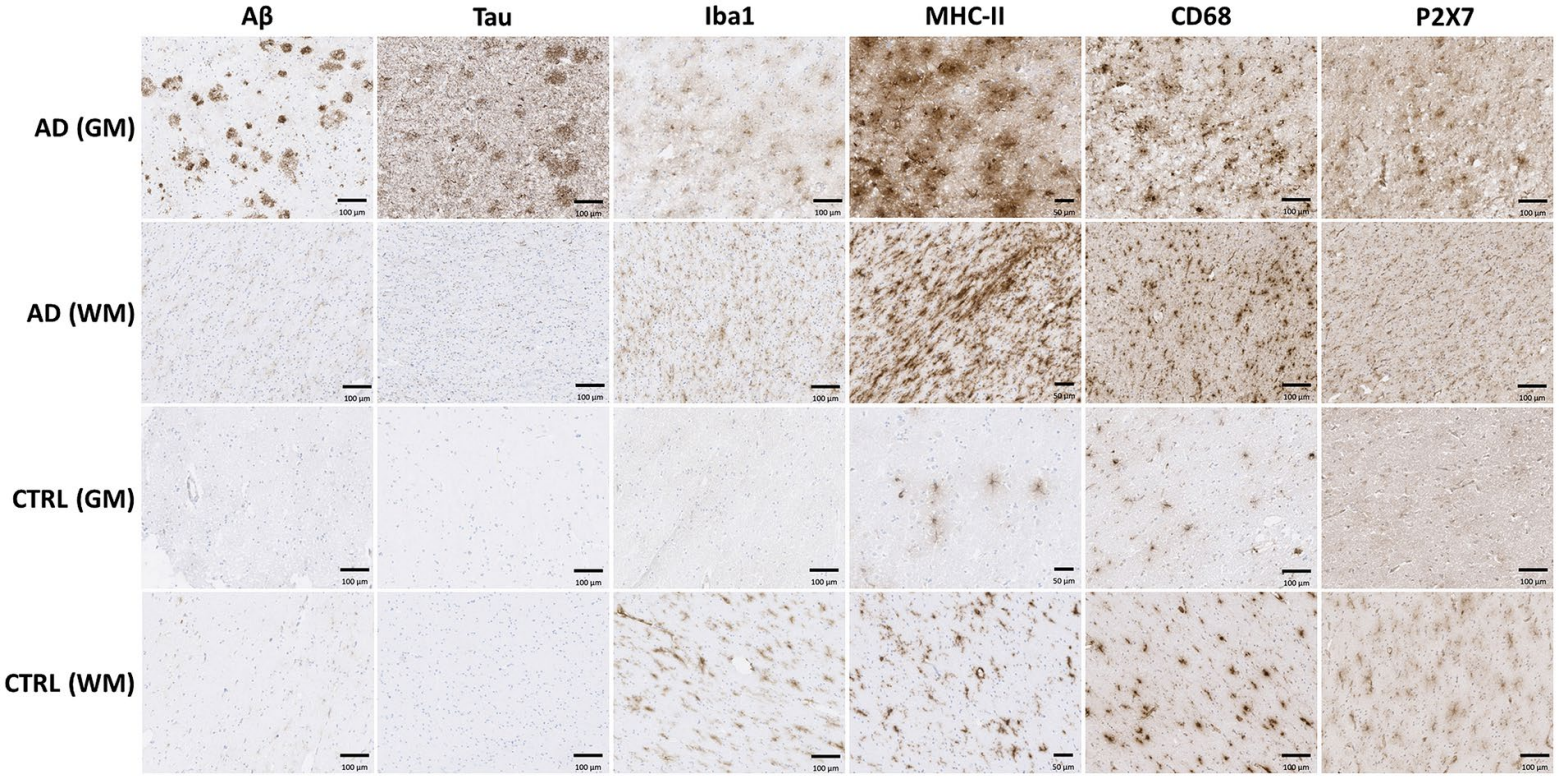
Representative images of immunohistochemical stainings on post mortem brain material of AD patients and non-neurological controls. Overall, staining for all markers is more pronounced in AD patients compared with non-neurological controls (CTRL), particularly in grey matter (GM). Staining for P2X_7_R is slightly, but significantly increased in both GM and WM of AD patients vs. CTRL. Scale bars indicate 100 µm, except in MHC-II images (50 µm).

Cryosections of post mortem brain of human AD patients (7 patients, temporal cortex, Braak stage 5–6) and post mortem age-matched non-neurological controls (CTRL, 7 subjects, temporal cortex, Braak stage 0–1) were incubated with [^11^C]SMW139 (28 nM), as well as with [^11^C]SMW139 together with JNJ-47965567 (10 µM; Fig. 7). To be able to correlate binding of [^11^C]SMW139 to (activated) microglia and plaque formation, adjacent sections were immunohistochemically stained for respectively P2X_7_R, Iba1, MHC-II, CD68 and amyloid β (Aβ) and tau (Fig. 8).

**Figure 9:**
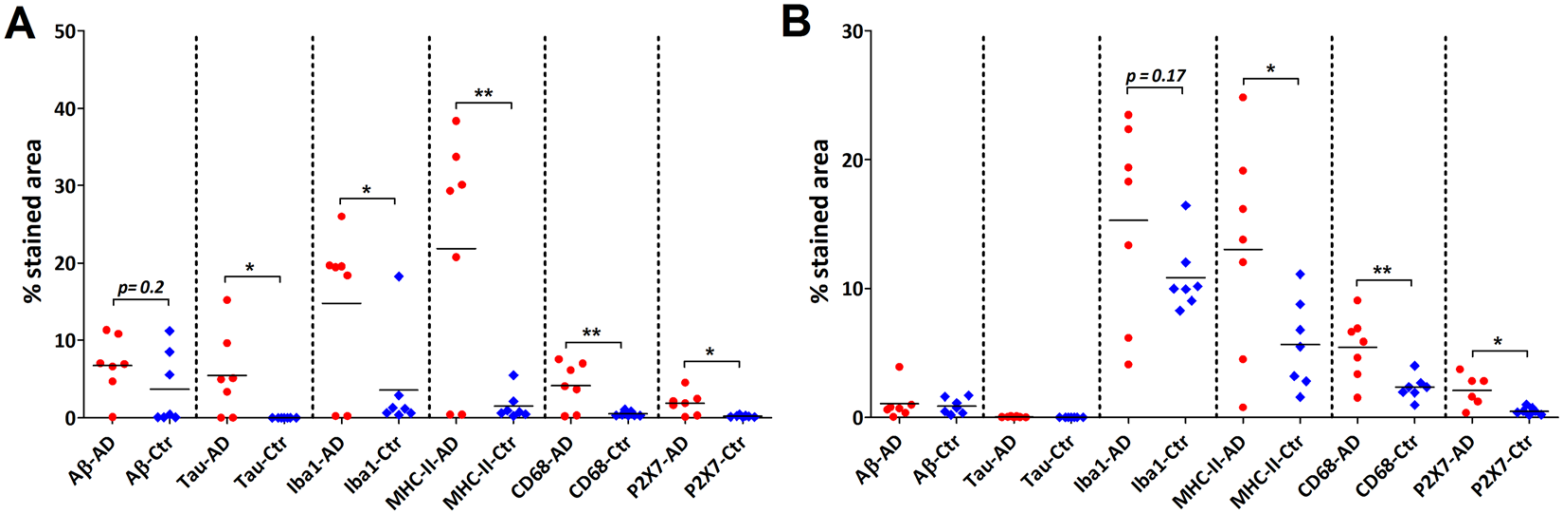
Quantification of immunohistochemical staining in human post mortem brain tissue. **(A**) Quantification of immunohistochemical staining in grey matter depicted per marker (Aβ, tau, Iba1, MHC-II, CD68 and P2X_7_R). Levels of pathological markers Aβ and tau were higher in AD patients (red circles) than in controls (blue diamonds) Levels of microglial markers Iba1, MHC-II, CD68 and P2X_7_R were significantly higher in AD patients compared with controls. *p < 0.05, **p < 0.01. (**B**) Quantification of immunohistochemical staining in white matter depicted per marker (Aβ, tau, Iba1, MHC-II, CD68 and P2X_7_R). Levels of microglial markers MHC-II, CD68 and P2X_7_R were significantly higher in AD patients (red circles) compared with controls (blue diamonds). *p < 0.05, **p < 0.01.

**Figure 10:**
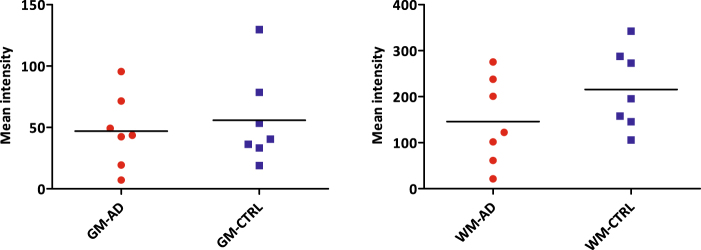
Quantification of binding of [^11^C]SMW139 in autoradiography experiments with human post mortem brain tissue. Both in grey (left) and white (right) matter, no significant differences were observed between AD patients (red circles) and non-neurological controls (blue squares).

Quantification of immunohistochemical staining showed significantly increased levels of the microglial markers Iba1, MHC-II, CD68 and P2X_7_R in both white and grey matter of AD patients (Fig. 9) vs. controls. As expected, immunohistochemical positive staining for tau and Aβ was only found in grey matter of AD patients. Immunohistochemical staining for P2X_7_R correlated with Iba1 (r^2^ = 0.7934) and MHC-II (r^2^ = 0.9082) staining in white matter of AD patients. In grey matter of both AD patients and controls, no correlation was found between P2X_7_R and any other marker. In autoradiography experiments, binding of [^11^C]SMW139 did not differ significantly between AD patients and controls (Fig. 10), neither in grey nor white matter, and in both AD patients and controls, tracer binding was higher in white matter than in grey matter. Although visually binding of [^11^C]SMW139 correlated with immunohistochemical staining for P2X_7_R, quantification showed no significant correlation between tracer binding and immunohistochemical staining.

## Discussion

Fluorinated analogues of adamantyl benzamide **1** were designed for higher brain uptake and enhanced metabolic stability *in vivo*^10^. To assess biodistribution and metabolism of these analogues *in vivo*, desmethyl precursors **5–8** were successfully radiolabelled via carbon-11 methylation to obtain [^11^C]**1–3** and [^11^C]SMW139 in good radiochemical yields with excellent radiochemical purity and with high molar activity. *Ex vivo* biodistribution studies in male Wistar rats showed no key differences between the four analogues, and all four analogues entered the brain. Fluorination at the bridgeheads of the adamantane moiety was suspected to block hydroxylation of the adamantane *in vivo*, and therefore increase metabolic stability, as was shown in a rat liver microsome assay (half-lives of 8, 5, 18 and 47 min for **1**, **2**, **3** and SMW139 respectively)^10^. Indeed, [^11^C]SMW139 was also found to have a lower fraction of radiometabolites in plasma *in vivo* (42 ± 2% of intact tracer at 45 min p.i.) in male Wistar rats. Interestingly, amounts of intact tracer in brain were significantly higher for both [^11^C]**1** and [^11^C]SMW139, compared with [^11^C]**2** and [^11^C]**3**. Radiometabolites were not identified for any of the analogues, but using the MetaPrint2D-React metabolite predictor^16,17^, possible radiometabolites could be formed by hydroxylation of the aromatic ring or cleavage at the amide bond. However, as all four analogues have these properties in common, it is unlikely that these would explain the observed differences in metabolism, which are therefore probably solely attributable to the adamantane moiety. The presence of a brain penetrant radiometabolite may be a limitation for the clinical use of [^11^C]SMW139, as this could hamper proper quantification. Despite optimisation of the extraction method used, the percentage of radioactivity that could be extracted from brain homogenates was on average only 60 ± 3% for all compounds. In addition, due to low expression of P2X_7_R in healthy brain, all analogues show rapid washout, leading to a low level of radioactivity in brain at 15 and 45 min p.i., and thus quantification of radiometabolites in brain is less accurate than that in plasma. Therefore, based on the highest amount of intact tracer in plasma, [^11^C]SMW139 was selected for further evaluation.

A rat model overexpressing human P2X_7_R, recently developed by Ory *et al*.^6^, is an excellent model to validate *in vitro* and *in vivo* tracer binding to the human receptor in a preclinical setting. The amount of binding of [^11^C]SMW139 that could be blocked in *in vitro* autoradiography studies using rAAV-*h*P2X_7_R injected rat brains, differed between the two compounds that were used for blocking. Where JNJ-47965567 blocked virtually all [^11^C]SMW139 binding to the rAAV-*h*P2X_7_R injected striatum, A-740003 only blocked 79 ± 10% of [^11^C]SMW139 binding. This can be explained by different binding sites for A-740003 versus [^11^C]SMW139 and JNJ-47965567. Whereas A-740003 is an orthosteric antagonist that binds to the ATP binding site of the receptor, [^11^C]SMW139 and JNJ-47965567 are allosteric antagonists^10,15^. Interestingly, the partial blocking of [^11^C]SMW139 observed with A-740003 suggests that both binding pockets have a significant overlap, a finding already proposed before by Bhattacharya *et al*.^15^.

*In vivo* PET in the same rat model revealed a 1.5-fold increase of [^11^C]SMW139 binding in the rAAV_3flag-*h*P2X_7_R injected striatum compared with the rAAV_3flag-eGFP injected striatum. This ratio was stable over time (Figure S5; Supplementary Information) and peaked within 15 min after tracer injection, suggesting good pharmacokinetic properties for a carbon-11 labelled PET tracer. Data obtained with [^11^C]SMW139 are comparable with those recently reported by Ory *et al*. using [^11^C]JNJ-54173717 in the same rat model^6^. Pre-treatment with JNJ-47965567 significantly decreased binding of [^11^C]SMW139 in the rAAV_3flag-*h*P2X_7_R injected striatum, showing specific binding of [^11^C]SMW139 to the *h*P2X_7_ receptor. Complete blocking also suggests either a high percentage of intact [^11^C]SMW139 left throughout the scanning time, as already indicated by the metabolite analysis, or the presence of a metabolite with affinity for P2X_7_R. The latter may also explain the difference in ipsi- to contralateral ratio between PET (1.5-fold) and AR experiments (10-fold), although to a lesser extent, this difference between *in vitro* and *in vivo* experiments is also seen with [^11^C]JNJ-54173717^6^. In an extended single microdose toxicity study in both male and female rats, no adverse or toxic effects of SMW139 (0.12 mg·kg^−1^) were observed, which allows for further evaluation of [^11^C]SMW139 in a clinical setting.

Results of the immunohistochemical staining of post mortem human brain tissue were as expected for Aβ and tau. Aβ staining showed disease related depositions in AD and diffuse Aβ deposits were observed in non-neurological control cases. Tau staining was present in grey matter of AD patients compared with absence of staining in non-neurological controls, which is in line with previous findings in AD pathology^18^. Staining for Iba1, a general marker for microglia, was not significantly different between AD and controls in white matter. Increased Iba1 staining was however observed in AD grey matter, as well as increased staining for activated microglia markers MHC-II and CD68. The latter two follow the same trend in AD white matter, suggesting a concerted neuroinflammatory reaction. Autoradiography experiments with [^11^C]SMW139 on human post mortem brain tissue did not show a statistically significant difference between AD patients and non-neurological controls, whereas immunohistochemical staining for P2X_7_R showed slightly increased expression in AD patients. The inability of [^11^C]SMW139 to show this difference may be explained by the lower spatial resolution that can be obtained in autoradiography experiments (25 µm) compared with that of immunohistochemical staining on a cellular level, in combination with the non-focal character of neuroinflammation in AD. Therefore, AD is probably not the appropriate neurological disease to study [^11^C]SMW139 in a clinical setting. However, although no statistical correlation was found, immunohistochemical staining for P2X_7_R seemed to overlap with binding of [^11^C]SMW139, which was confirmed by the virtually complete blocking of tracer binding with the selective, non-structurally related P2X_7_R antagonist JNJ-47965567^15^. In general, higher tracer binding was observed in WM compared with GM in the human brain sections. The high level of binding in WM may be explained by the higher abundance of microglia in WM compared with GM^19^, which would in turn explain higher P2X_7_R expression in WM, as the receptor is mainly expressed in microglia cells^2^. This effect was not observed in PET images in rats, however, differences in binding of [^11^C]SMW139 were also much less pronounced in autoradiography experiments between rat WM and rat GM compared with the human situation.

Despite these somewhat disappointing results *in vitro* for AD, recent results of Territo *et al*. with [^11^C]GSK1482160^7^ and Fantoni *et al*. with [^18^F]EFB^8^, both showing increased P2X_7_R targeted tracer uptake in brain, after systemic administration of LPS in rodents, indicates the involvement of P2X_7_R in microglial activation. However, it should be noted that currently knowledge of actual P2X_7_R expression levels in human is lacking, both in health and disease. Therefore, the usefulness of P2X_7_R as a target in PET imaging of microglial activation remains to be elucidated. This is hampered by differences between rat and human P2X_7_ receptor, often resulting in a lower affinity of compounds for the rodent receptors compared with the human receptor, complicating preclinical evaluation of tracers targeting P2X_7_R. However, using [^3^H]A-740003, a non-brain penetrant P2X_7_R antagonist with similar affinities for rat (18 nM) and human P2X_7_R (40 nM)^12,13^, increased P2X_7_R expression was recently shown to be highest at the peak of the disease in a rat model of MS *in vitro*^20^. In line with that, using the same tracer in human brain sections of MS patients, high P2X_7_R expression was found in active MS lesions. As the affinity of [^11^C]SMW139 is 6-fold lower for mouse P2X_7_R compared with *h*P2X_7_R^10^, evaluation of [^11^C]SMW139 on post mortem brain tissue of patients diagnosed with MS would be interesting to validate the potential of this tracer.

In conclusion, [^11^C]SMW139 binds specifically to P2X_7_R *in vivo* in a rat model overexpressing the human P2X_7_ receptor. In addition, as a first step in translation to human application, SMW139 showed no toxic effects in an extended single microdose toxicity study in rats. Although *in vitro*, in brain tissue of Alzheimer’s disease patients no significant difference in P2X_7_R expression was observed compared with brain tissue of healthy controls, recent results using different tracers targeting P2X_7_R in animal models of neuroinflammation and MS^7–9,20^ indicate the involvement of the receptor in specific neuroinflammatory conditions. [^11^C]SMW139 could therefore be a promising PET tracer for P2X_7_R imaging in microglial activation and warrants further investigation in other diseases involving neuroinflammation, particularly in those in which an increased expression of P2X_7_R is expected. Clinical validation of [^11^C]SMW139 in MS patients is currently ongoing.

## Methods

### Chemistry

Synthesis of precursors **5**–**8** was performed either by demethylation (**5**) or a PyBOP coupling (**6**–**8**) between 2-chloro-5-hydroxybenzoic acid **12** and the appropriate adamantan-1-ylmethylamine and is described in detail in the Supplementary Information.

### Radiosynthesis

[^11^C]**1**–**3** and [^11^C]SMW139 were obtained by reaction of the appropriate precursor (**5**–**8**) with [^11^C]methyl iodide in the presence of NaOH for 4 min at 85 °C in an overall synthesis time of 35–40 min. Radiosynthesis, including purification, formulation and analysis is described in detail in the Supplementary Information.

LogD_oct,7.4_ of [^11^C]**1**–**3** and [^11^C]SMW139 was determined by the shake-flask method as described in the Supplementary Information.

### *In vivo* evaluation in healthy rats

For each of the carbon-11 labelled benzamide analogues [^11^C]**1**–**3** and [^11^C]SMW139, a biodistribution study and metabolite analysis in healthy male Wistar rats was performed, as described in detail in the Supplementary Information. Briefly, tracer distribution in blood, brain, and peripheral organs was measured at 5, 15, 30 and 45 min after intravenous injection of 13–23 MBq of formulated tracer. Metabolite analysis was performed at 15 and 45 min after intravenous injection of 30–40 MBq of formulated tracer in both blood plasma and brain.

### *In vitro* autoradiography

Snap frozen brain tissue was cut on a cryostat into 20 μm sections. Sections were incubated with either [^11^C]SMW139 (28 nM) alone, or tracer in the presence of a blocking compound: A-740003 (10 μM) or JNJ-47965567 (10 μM). Autoradiography experiments are described in detail in the Supplementary Information.

### Immunohistochemical staining

Frozen brain tissue was cut into section of 5 μm and fixed with acetone. The following primary antibodies were used: Mouse monoclonal anti-phospho-tau (AT8 for tau pSer202 and pThr205, 1:800, Pierce Biotechnology), mouse monoclonal anti-Aβ (IC16 1:200, Prof. C. Korth, Heinrich Heine University Düsseldorf, Germany), Rabbit polyclonal anti-Iba1 (1:3200, Wako), mouse monoclonal anti–MHC class II (CR3/43, 1:200, Dako), mouse monoclonal anti-CD68 (KP1, 1:600, Dako), and rabbit polyclonal anti-P2X7 (ab77413, 1:600, Abcam). Further details of the immunohistochemical staining procedure can be found in the Supplementary Information.

### PET imaging in rats locally overexpressing *h*P2X_7_R

Adeno-associated viral (AAV) vectors overexpressing *h*P2X_7_ and *e*GFP were constructed and produced as described by Ory *et al*.^6^. Female rats were injected with the *h*P2X_7_R expressing vector in the right striatum, and *e*GFP expressing vector in the left striatum, which is described in detail in the Supplementary Information. Rats were sacrificed for *in vitro* autoradiography at 3 (n = 2) and 12 (n = 5) weeks after vector injection. Scanning experiments were performed at 5 and 11 weeks after viral vector injection. Stable expression of *r*AAV vectors in rat brain has been previously demonstrated between 5 and 10 weeks after vector expression^6,21^. To confirm stable expression of *h*P2X_7_R during the time period in which PET scanning studies were conducted, autoradiography experiments were performed both prior to and following PET experiments. In a dedicated small animal PET scanner (nanoPET, Mediso Ltd.), 60 min dynamic PET scans were acquired immediately after intravenous administration of 16–20 MBq of [^11^C]SMW139. For blocking experiments, rats were injected subcutaneously with JNJ-47965567 (30 mg·kg^−1^)^14,15^ 45 min prior to tracer injection. After data reconstruction (details in Supplementary Information), images were analysed using the freely available AMIDE software (version 0.9.2; http://amide.sourceforge.net). ROIs were drawn around ipsi- and contralateral striata. Results are expressed as standardised uptake values (SUVs) and error bars indicate standard deviation.

### Extended single microdose toxicity study

A single microdose toxicity study for P2X_7_R antagonist SMW139 was performed according to EMEA guideline EMA/CPMP/ICH/286/1995 (December 2009) in healthy Wistar rats (male and female) at a dose of 0.12 mg·kg^−1^. Rats were administered SMW139 or vehicle and parameters assessed post-dose were clinical signs and mortality, changes in body weight, food consumption, terminal haematology, coagulation and clinical chemistry parameters, gross pathology and organ weights. Further details are described in the Supplementary Information.

### Ethics approval

All animal studies were conducted in accordance with the European Community Council Directive 2010/63/EU or UK Home Office regulations for laboratory animal ethics and welfare and experiments were approved by the animal ethics committees of local authorities (Dierexperimentencommissie (DEC) of the VU and VUmc Amsterdam; KU Leuven University Ethics Committee for Animals; UK Home Office and Animal Welfare and Ethical Review Body at Pharmidex Ltd., London, UK).

Human brain tissue samples from Alzheimer’s disease patients and non-neurological control cases were obtained from The Netherlands Brain Bank (NBB, www.brainbank.nl), Netherlands Institute for Neuroscience (Amsterdam, the Netherlands). The NBB’s procedures are in accordance with all national laws and regulations and have been approved by the Medical Ethics Committee of the VU University Medical Center (Amsterdam, the Netherlands). All donors or their next of kin gave written informed consent for a brain autopsy and the use of the material and clinical information for research purposes. All tissue samples were handled according to Dutch national ethical guidelines (Code for Proper Secondary Use of Human Tissue, Dutch Federation of Medical Scientific Societies).

### Data availability

The data generated during and/or analysed during the current study are available from the corresponding author on reasonable request.

## Electronic supplementary material

Supplementary Information
