# Supplementary material for: Identification of the allosteric P2X7 receptor antagonist [11C]SMW139 as a PET tracer of microglial activation
Source: Sci Rep. 2018 Apr 26;8:6580. doi: 10.1038/s41598-018-24814-0 (PMC5920098; doi:10.1038/s41598-018-24814-0)
Supplement: Supplementary file 1 — Supplementary Information [file 41598_2018_24814_MOESM1_ESM.pdf]

Supplementary information

Identification of the allosteric P2X$_7$ receptor antagonist [^{11}C]SMW139 as a PET tracer of microglial activation

Bieneke Janssen[a], Danielle J. Vugts[a], Shane M. Wilkinson[b], Dieter Ory[c], Sylvie Chalon[d], Jeroen J.M. Hoozemans[e], Robert C. Schuit[a], Wissam Beaino[a], Esther J.M. Kooijman[a], Johan J. van den Hoek[a], Mansoor Chishty[f], Aurélie Doméné[d], Anke Van der Perren[g], Alessandro Villa[h], Adriana Maggi[h], Ger T. Molenaar[a,i], Uta Funke[a,c,i], Rostislav V. Shevchenko[f], Veerle Baekelandt[g], Guy Bormans[c], Adriaan A. Lammertsma[a], Michael Kassiou[b], Albert D. Windhorst[a]

[a] Department of Radiology & Nuclear Medicine, Neuroscience Campus Amsterdam, VU University Medical Center, Amsterdam, the Netherlands

[b] School of Chemistry, University of Sydney, Sydney, Australia

[c] Laboratory for Radiopharmaceutical Research, Department of Pharmaceutical and Pharmacological Sciences, KU Leuven, Leuven, Belgium

[d] UMR 1253, iBrain, Université de Tours, Inserm, Tours, France

[e] Department of Pathology, VU University Medical Center, Amsterdam, the Netherlands

[f] Pharmidex Pharmaceutical Services Ltd., London, United Kingdom

[g] Neurobiology and Gene Therapy, Department of Neurosciences, KU Leuven, Leuven, Belgium

[h] Center of Excellence on Neurodegenerative Diseases and Department of Pharmacological and Biomolecular Sciences, University of Milan, Milan, Italy

[i] BV Cyclotron VU, Amsterdam, the Netherlands

Corresponding authors: Bieneke Janssen, b.janssen@vumc.nl

Albert D. Windhorst, ad.windhorst@vumc.nl

Material and Methods

Chemistry

General

All solvents and reagents were obtained from commercial sources, and dried and purified when necessary according to published standard procedures [1]. Reactions were performed at room temperature unless stated otherwise.

Analytical thin layer chromatography (TLC) was performed using 0.2 mm thick, aluminum-backed, pre-coated silica gel plates (Merck Silica gel 60 F_{254}). Compounds were visualized by short and long wavelength ultra-violet fluorescence and by staining with potassium permanganate (40 g potassium carbonate, 6 g potassium permanganate, 600 mL H_2O then 5 mL of 10% NaOH).

Evaporation or concentration under reduced pressure refers to evaporation using a rotary evaporator connected to a vacuum pump. Removal of residual solvent, when necessary, was achieved by evacuation (0.01 - 0.1 mm Hg) with a high stage oil sealed vacuum pump.

Normal-phase flash chromatography was performed using Merck Silica gel 60 (230 – 400 mesh ASTM), under a positive pressure of N_2, with the indicated solvents. Solvent compositions were mixed volume per volume (*v*/*v*) as specified.

Melting points were determined using an Optimelt MPA100 (Lambda Photometrics Ltd, Hertfordshire, UK) automated melting point apparatus and are uncorrected. Elemental analysis was determined in duplicate using a PerkinElmer PE2400 Elemental Analyzer (CHNS) at Macquarie University, NSW, Australia, and are uncorrected.

Nuclear magnetic resonance (NMR) spectra were obtained using a Bruker (Billerica, MA, USA) DRX400 (400 MHz for ^{1}H and 100 MHz for ^{13}C and ^{19}F) or a Bruker AVANCE III 500 Ascend (500 MHz for ^{1}H and 126 MHz for ^{13}C and ^{19}F) at 300 K unless otherwise stated. ^{13}C and ^{19}F spectra were obtained with complete proton decoupling unless otherwise stated. Chemical shift (™) data are expressed in ppm relative to ™$_{TMS}$ = 0, using deuterated solvent as an internal reference. Data are reported as ™, relative integral, observed multiplicity (s = singlet, d = doublet, dd = doublet of doublets, quart = quartet, quint = quintet, m = multiplet, br = broad), coupling constant(s) (*J* Hz) and assignment (unassigned

diastereotopic protons are allocated lower case letters e.g. a, b, etc.). All multiplicities and coupling constants are apparent. Assignment of signals was assisted by 2D multiplicity-edited HSQC and HMBC experiments where necessary.

Low resolution mass spectra (LRMS) were obtained using a Bruker AmaZon SL ion trap mass spectrometer with electro-spray ionization in either positive (+ESI) or negative (-ESI) mode. Atmospheric-pressure chemical ionization samples were run as a solid sample using the atmospheric solid analysis probe attachment. High resolution mass spectra (HRMS) was obtained from a Bruker Apex Qe 7T Fourier Transform Ion Cyclotron resonance mass spectrometer with electro-spray ionization in either positive (+ESI) or negative (-ESI) mode. Data are expressed as observed mass (*m/z*), assignment (M = molecular ion), and relative intensity (%).

High Performance Liquid Chromatography (HPLC) was performed using a Waters (Milford, MA, USA) 2695 Separations module equipped with a Waters Alliance Series Column Heater (set at 30 °C) and Waters 2996 Photodiode Array (PDA) Detector. Samples were resolved on a Waters SunfireTM C18 5 µm column (2.1 x 150 mm) using a gradient of water and acetonitrile. Acetonitrile was increased from 0 to 100% over 30 min at a flowrate of 0.2 mL·min^{-1}. Data acquisition and processing were performed using Waters Empower 2 software. Samples were analyzed in duplicate.

General procedure precursor synthesis

(Benzotriazol-1-yloxy)tripyrrolidinophosphonium hexafluorophosphate (PyBOP, 1.1 mmol) was added to a solution of appropriate adamantan-1-ylmethylamine (1.0 mmol), 2-chloro-5-hydroxybenzoic acid **12** (1.1 mmol), and diisopropylethylamine (1.75 mmol) dissolved in *N*,*N*-dimethylformamide (10 mL). The reaction was stirred overnight (18 h) at room temperature under a nitrogen atmosphere. The reaction was diluted with a 10% methanol in dichloromethane solution (10 mL) and then washed with aqueous hydrochloric acid (1 M, 10 mL). The organic layer was put aside and the aqueous layer was extracted with 10% methanol in dichloromethane solution (2 x 2 mL). The organic layers were combined, dried ($MgSO_4$), filtered and evaporated to dryness by rotary evaporation. The resulting residue was subjected to flash chromatography (1:19 methanol:dichloromethane) to yield *N*-(adamantan-1-ylmethyl)-2-chloro-5-hydroxybenzamides.

N-(Adamantan-1-ylmethyl)-2-chloro-5-hydroxybenzamide (5).

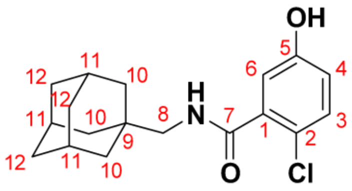

A solution of boron tribromide in dichloromethane (1M, 3.0 mL, 3.0 mol) was added to a solution of *N*-(adamantan-1-ylmethyl)-2-chloro-5-methoxybenzamide **1** (300 mg, 0.90 mmol) in dichloromethane (16 mL) at 0 °C. The reaction was warmed to room temperature and stirred for 2 days. The reaction was quenched with methanol (6 mL) and the solvent removed by rotary evaporation. The residue was diluted in ethyl acetate (150 mL) and washed with water (3 x 50 mL). The organic layer was dried over $MgSO_4$ and evaporated to dryness by rotary evaporation. The light brown solid was subjected to flash chromatography (1:19 methanol:dichloromethane) to yield N-(adamantan-1-ylmethyl)-2-chloro-5-hydroxybenzamide **5** (288 mg, quant.) as a colourless solid. Further purification could be obtained through recrystallisation from acetone/water.

R_f 0.28 (1:19 MeOH:CH_2Cl_2); **M.P.** 264-266 °C (decomp.); **^{1}H NMR** (500 MHz, d_6-DMSO): δ 9.84 (1H, s, OH), 8.23 (1H, t, $^3J_{NH\text{-}H8}$ = 6.3 Hz, NH), 7.23 (1H, d, $^3J_{H3\text{-}H4}$ = 8.5 Hz, H3), 6.79 (1H, dd, $^3J_{H4\text{-}H3}$ = 8.5 Hz, $^4J_{H4\text{-}H6}$ = 2.5 Hz, H4), 6.77 (1H, d, $^3J_{H6\text{-}H4}$ = 3.0 Hz, H6), 2.90 (2H, $^3J_{H8\text{-}NH}$ = 6.0 Hz, H8), 1.94 (3H, br s, H11), 1.63 (6H, m, H12), 1.51 (6H, s, H10) ppm; **^{13}C NMR** (126 MHz, d_6-DMSO): δ 166.5 (C7), 156.0 (C5), 138.2 (C1), 130.3 (C3), 119.0 (C2), 117.3 (C4), 115.5 (C6), 50.4 (C8), 39.9 (C10), 36.5 (C12), 34.1 (C9), 27.7 (C11) ppm; **LRMS** (+ESI): 661 ($[2M+Na]^+$, 100), 342 ($[M+Na]^+$, 16), 320 ($[M+H]^+$, 9); **HRMS** (+ESI) Calc. for $C_{18}H_{22}{}^{35}ClNO_2$ $[M+Na]^+$: 342.1234, found: 342.1232; **Anal.** ($C_{18}H_{22}ClNO_2$): calc, C 67.60, H 6.93, N 4.38; found, C 67.78, H 7.04, N 4.11.

2-chloro-*N*-((3-fluoroadamantan-1-yl)methyl)-5-hydroxybenzamide (6)

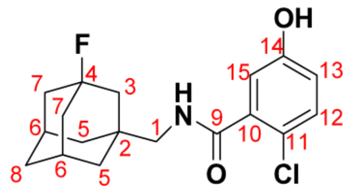

(3-Fluoroadamantan-1-yl)methanamine **9** (137 mg, 0.748 mmol) and 2-chloro-5-hydroxybenzoic acid **12** (142 mg, 0.822 mmol) were coupled using the **general procedure** to obtain 2-chloro-*N*-((3-fluoroadamantan-1-yl)methyl)-5-hydroxybenzamide **6** (184 mg, 73%) as a colourless solid.

M.P. 235-236 °C; **IR** (Di-ATR): 3324 (N-H), 3116 (br, O-H), 2917 (C-H), 2852 (C-H), 1624 (C=O), 1568, 1474, 1277, 1224 (Ar-O), 1021 (C-F), 827, 711, 646, (C-Cl), 636, 539 cm^{-1}; **^{1}H NMR** (500 MHz, d_6-DMSO): δ 9.85 (1H, s, OH), 8.33 (1H, t, $^3J_{NH\text{-}H1}$ = 6.3 Hz,), 7.24 (1H, d, $^3J_{H12\text{-}H13}$ = 9.0 Hz, H12), 6.80 (1H, dd, $^3J_{H13\text{-}}$

$_{H12}$ = 8.5 Hz, $^4J_{H13-H15}$ = 3.0 Hz, H13), 6.77 (1H, d, $^4J_{H15-H13}$ = 3.0 Hz, H15), 3.02 (2H, d, $^3J_{H1-NH}$ = 6.5 Hz, H1), 2.24 (2H, br s, H6), 1.76 (4H, m, H7), 1.64 (2H, d, $^3J_{H3-F}$ = 5.5 Hz, H3), 1.57-1.38 (6H, m, H5+H8) ppm; **^{13}C NMR** (126 MHz, d_6-DMSO): δ 166.6 (s, C9), 156.1 (s, C14), 138.1 (s, C10), 130.4 (s, C12), 118.9 (s, C11), 117.4 (s, C13), 115.4 (s, C15), 92.9 (d, $^1J_{C4-F}$ = 182.3Hz, C4), 49.1 (s, C1), 44.9 (d, $^3J_{C3-F}$ = 17.6 Hz, C3), 41.8 (d, $^2J_{C7-F}$ = 17.6 Hz, C7), 39.1 (d, $^3J_{C2-F}$ = 9.3 Hz, C2), 38.3 (s, C5), 34.7 (s, C8), 30.5 (d, $^3J_{C6-F}$ = 8.8 Hz, C6) ppm; **^{19}F NMR** (471 MHz, d_6-DMSO): δ -128.3 (s, C4-F) ppm; **LRMS** (+ESI): 699 ([2M+Na]$^+$, 63), 697 ([2M+Na]$^+$, 100), 360 ([M+Na]$^+$, 16), 338 ([M+H]$^+$, 24); **HRMS** (+ESI) Calc. for $C_{18}H_{22}{}^{37}ClFNO_2$ [M+H]$^+$: 340.1288, found: 340.1293; Calc. for $C_{18}H_{22}{}^{35}ClFNO_2$ [M+H]$^+$: 338.1318, found: 338.1323.

2-chloro-*N*-((3,5-difluoroadamantan-1-yl)methyl)-5-hydroxybenzamide (7)

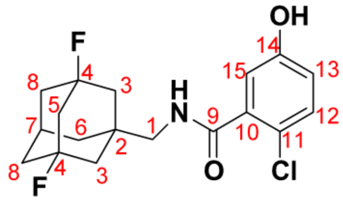

3,5-Difluoroadamantan-1-yl)methanamine **10** (334 mg, 1.66 mmol) and 2-chloro-5-hydroxybenzoic acid **12** (315 mg, 1.83 mmol) were coupled using the **general procedure** to obtain 2-chloro-*N*-((3,5-difluoroadamantan-1-yl)methyl)-5-hydroxybenzamide **7** (455 mg, 77%) as a colourless solid.

M.P. 232-234 °C; **IR** (Di-ATR): 3325 (N-H), 3101 (br, O-H), 2926 (Ar-H), 2861 (C-H), 1625 (C=O), 1567, 1473, 1328 (Ar-O), 997 (C-F), 828, 648, 635 (C-Cl), 546 cm^{-1}; **^{1}H NMR** (500 MHz, $CDCl_3$): δ 9.89 (1H, br s, OH), 8.42 (1H, t, $^3J_{NH}$ = 6.4 Hz, NH) 7.25 ppm (1H, d, $^3J_{H12-H13}$ = 8.6 Hz, H12), 6.81 (1H, dd, $^3J_{H13-H12}$ = 8.6 Hz, $^4J_{H13-H15}$ = 2.9 Hz, H13), 6.78 (1H, d, $^4J_{H15-H13}$ = 2.9 Hz, H15), 3.12 (2H, d, $^3J_{H1-NH}$ = 6.3 Hz, H1), 2.41 (1H, m, H7), 2.00 (2H, m, H5), 1.83-1.58 (8H, m, H3+H8), 1.36 (2H, br s, H6) ppm; **^{13}C NMR** (126 MHz, $CDCl_3$): δ 166.7 (s, C9), 156.1 (s, C14), 137.9 (s, C10), 130.4 (s, C12), 118.9 (s, C11), 117.4 (s, C13), 115.4 (s, C15), 93.3 (dd, $^1J_{C4-F}$ = 186.3 Hz, $^3J_{C4-C4'F}$ = 13.6 Hz, C4), 47.9 (s, C1), 47.0 (t, $^2J_{C5-F}$ = 18.8 Hz, C5), 43.6 (m, C3), 40.5 (t, $^3J_{C2-F}$ = 9.8 Hz, C2), 40.2-39.4 (m, C8, obstructed by solvent peak), 36.7 (s, C6), 30.0 (t, $^3J_{C7-F}$ = 10.5 Hz, C7) ppm; **^{19}F NMR** (471 MHz, $CDCl_3$): δ -133.3 (s, C4-F) ppm; **LRMS** (+ESI): 380 ([M+Na]$^+$, 40), 378 ([M+Na]$^+$, 100); **HRMS** (+ESI) Calc. for $C_{18}H_{21}{}^{37}ClF_2NO_2$ [M+H]$^+$: 358.1199, found: 358.1193; Calc. for $C_{18}H_{21}{}^{35}ClF_2NO_2$ [M+H]$^+$: 356.1229, found: 356.1222.

2-chloro-5-hydroxy-*N*-((3,5,7-trifluoroadamantan-1-yl)methyl)benzamide (8)

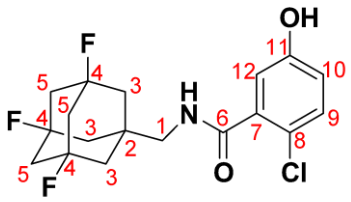

3,5,7-Trifluoroadamantan-1-yl)methanamine **11** (137 mg, 0.625 mmol) and 2-chloro-5-hydroxybenzoic acid **12** (119 mg, 0.687 mmol) were coupled using the **general procedure** to obtain 2-chloro-*N*-((3,5-difluoroadamantan-1-yl)methyl)-5-hydroxybenzamide **8** (184 mg, 79 %) as a colourless solid.

M.P. 230-231 °C; **IR** (Di-ATR): 3427 (N–H), 3266 (O–H), 2962 (C–H), 1649 (C=O), 1540 (C=C), 1338 (C–F), 1025 (C–O) cm^{-1}; ^{1}**H NMR** (400 MHz, DMSO-d_6): δ 9.90 (1H, br s, OH), 8.49 (1H, br t, $^{3}J_{NH\text{-}H1}$ = 6.3 Hz, NH), 7.26 (1H, dt, $^{3}J_{H9\text{-}H10}$ = 8.7 Hz, *J* = 1.3, H9), 6.90-6.75 (2H, m, H10, H12), 3.22 (2H, d, $^{3}J_{H1\text{-}NH}$ = 6.3 Hz), 2.22–2.09 (3H, m, H5a), 2.06–1.93 (3H, m, H5b), 1.68 (6H, br s, H3) ppm; ^{13}**C NMR** (101 MHz, DMSO-d_6): δ 166.8 (s, C6), 156.2 (s, C11), 137.8 (s C7), 130.4 (s, 9), 118.8 (s, C8), 117.5 (s, C10), 115.4 (s, C12), 92.7 (dt, $^{1}J_{C4\text{-}F}$ = 188.3, $^{3}J_{C4\text{-}C4'F}$ = 15.3 Hz), 47.0 (d, $^{4}J_{C1\text{-}F}$ = 1.6 Hz, C1), 45.9–45.1 (m, C5), 42.6–42.0 (m, C3), 38.2 (q, $^{3}J_{C2\text{-}F}$ = 10.9 Hz, C2) ppm; ^{19}**F NMR** (376 MHz, DMSO-d_6): δ –140.4 ppm; **LRMS** (+ESI) m/z: 396 ($[M+Na]^+$, 100%), 769 ($[2M+Na]^+$, 20%); **Anal.** Calcd for $C_{18}H_{19}ClF_3NO_2$: C, 57.84; H, 5.12; N, 3.75. Found: C, 57.88; H, 5.14; N, 3.65; **HPLC:** 99.2%, t_R: 21.04 min.

Radiosynthesis

General

Chemicals and solvents were obtained from commercial sources and used as received, unless stated otherwise. Analytical isocratic HPLC was performed using a Jasco (Easton, MD, USA) PU-1580 station with a Jasco UV-2075 Plus UV detector (285 nm) and a NaI radioactivity detector (Raytest, Straubenhardt, Germany). Chromatograms were acquired using Raytest GINA Star software (version 5.01). Semi-preparative isocratic HPLC was performed using a Jasco PU-1587 station with a Jasco UV-1575 UV detector (254 nm), a custom-made radioactivity detector and chromatograms were acquired using Jasco ChromNAV CFR software (version 1.14.01).

N-((adamantan-1-yl)methyl)-2-chloro-5-[^{11}C]methoxybenzamide ([^{11}C]1)

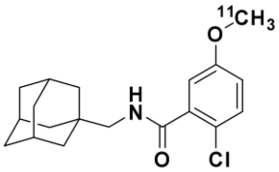

[^{11}C]CO_2 was produced by a ^{14}N(p,α)^{11}C nuclear reaction performed in a 0.5% O_2/N_2 gas mixture using an IBA Cyclone 18/9 cyclotron (IBA, Louvain-la-Neuve, Belgium). Subsequently, [^{11}C]CO_2 was transferred to an in-house built synthesis unit and trapped in a reaction vial containing 0.1 mL of 0.1 M $LiAlH_4$ in THF. After evaporation of THF by heating the reaction vial to 130 °C under a helium flow, 0.25 mL of 60% HI in water was added. [^{11}C]CH_3I was then distilled to a reaction vial containing a solution of precursor **5** (0.5 mg, 1.5 µmol) and sodium hydroxide (NaOH) (10 µL, 5 µmol, 0.5 M solution (10% water in DMSO)) in 0.2 mL of DMSO. The reaction mixture was heated at 85 °C for 4 min, after which it was diluted with 1 mL of water and purified by HPLC on a Reprospher C18-DE 5 µm (50 x 8 mm) column (Dr. Maisch, Ammerbuch-Entringen, Germany) using 50 mM NH_4OAc (pH 10)/ethanol (EtOH) (45:55, *v/v*) as eluent at a flow rate of 3 mL·min^{-1}. The fraction containing [^{11}C]**1** (t_R = 10 min) was collected, and neutralised with 1 mL of citrate buffer (containing citric acid monohydrate (8 mg·mL^{-1}), sodium citrate dehydrate (16 mg·mL^{-1}) and sodium acetate trihydrate (7 mg ·mL^{-1})) and diluted with 8-15 mL of 7.09 mM NaH_2PO_4 in saline (pH 5.2). [^{11}C]**1** was obtained in a radiochemical yield (RCY) of 33 ± 9% (n = 6), calculated from [^{11}]CO_2, with a radiochemical purity >97%, a molar activity (A_m) of 159 ± 77 GBq·µmol^{-1} (n = 6) at end of synthesis (EOS) and an overall synthesis time of 35-40 min. The identity of the product was confirmed by analytical HPLC using co-injection of the product and non-labelled **1** (Platinum C18 5 µm (4.6 x 250 mm) column (Grace, Columbia, MD, USA) using acetonitrile (MeCN)/H_2O/diisopropylamine (DIPA) (60:40:0.1, *v/v/v*) as eluent at a flow rate of 1 mL·min^{-1}, t_R = 6 min).

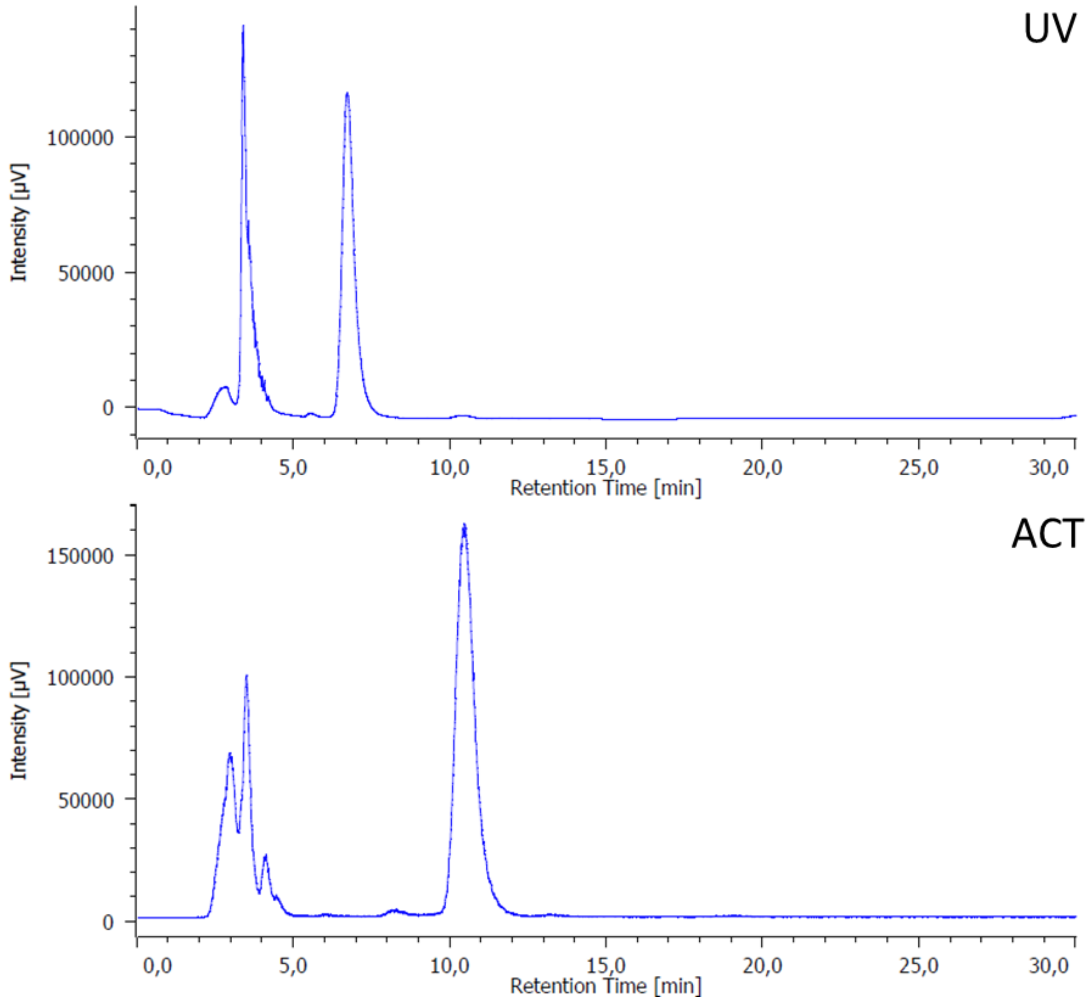

Preparative HPLC chromatogram of [^{11}C]1 (Reprospher C18-DE, 50 mM NH_4OAc (pH 10)/EtOH 45:55, 3 mL·min^{-1}, 254 nm).

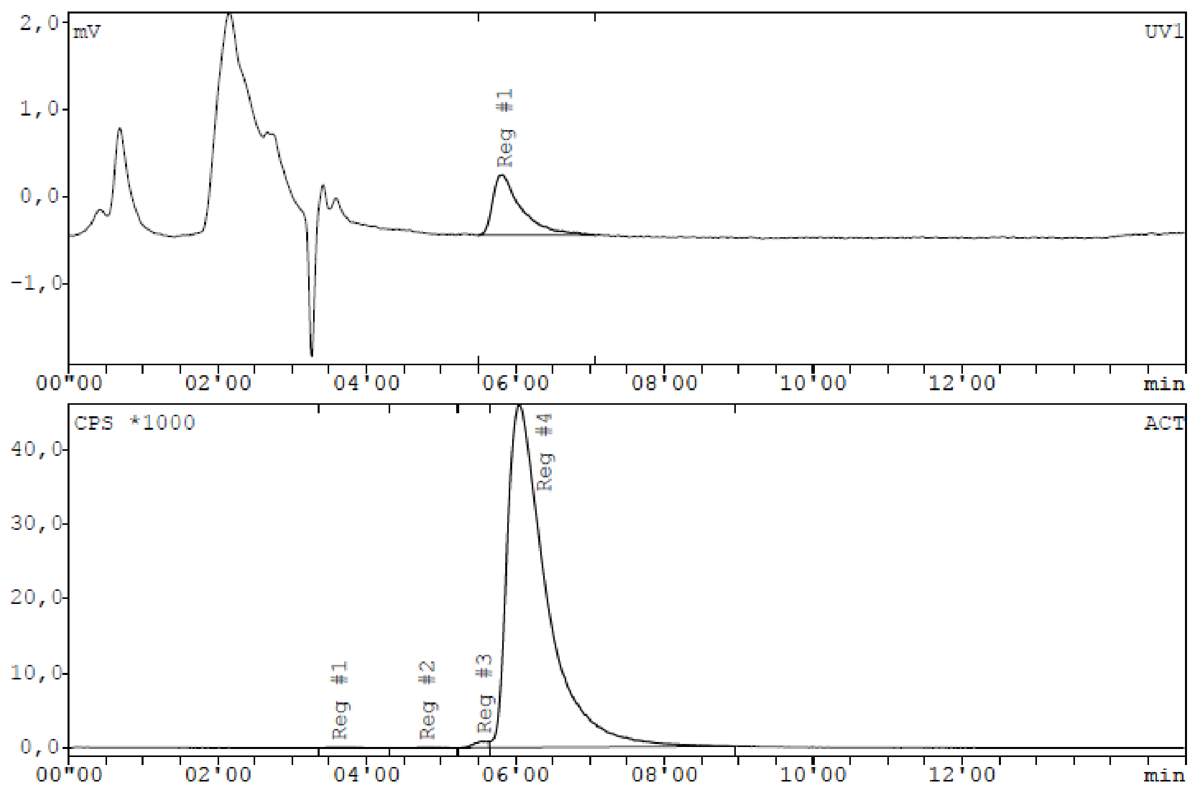

Analytical HPLC chromatogram of [^{11}C]1 formulation (Platinum C18, MeCN/H_2O/DIPA 50:50:0.1, 1 mL·min^{-1}, 285 nm).

2-chloro-*N*-((3-fluoroadamantan-1-yl)methyl)-5-[^{11}C]methoxybenzamide ([^{11}C]2)

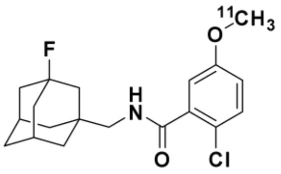

In a procedure analogous to that given above for [^{11}C]**1**, [^{11}C]**2** was obtained from precursor **6** (0.5 mg, 1.5 µmol). During preparative HPLC on a Reprospher C18-DE 5 µm (50 x 8 mm) column with MeCN/H_2O/DIPA (60:40:0.1, *v*/*v*/*v*) as eluent at a flow rate of 3 mL·min^{-1}, the fraction containing [^{11}C]**2** (t_R = 10 min) was collected, diluted with 40 mL of water and trapped on a Sep-Pak Plus tC18 cartridge (Waters, Milford, MA, USA), preconditioned with 1 mL of ethanol and 10 mL of water. The cartridge was washed with 20 mL of water and subsequently [^{11}C]**2** was eluted with 1 mL of EtOH and diluted with 10 mL of 7.09 mM NaH_2PO_4 in saline. [^{11}C]**2** was obtained in a RCY of 30 ± 8% (n = 5), calculated from [^{11}C]CO_2, with a radiochemical purity >97%, A_m of 58 ± 7 GBq·µmol^{-1} (n = 5) at EOS and an overall synthesis time of 35-40 min. The identity of the product was confirmed by analytical HPLC using co-injection of the product

and non-labelled **2** (Platinum C18 5 µm (4.6 x 250 mm) column using MeCN/H_2O/DIPA (50:50:0.1, *v*/*v*/*v*) as eluent at a flow rate of 1 mL·min^{-1}, t_R = 6.4 min).

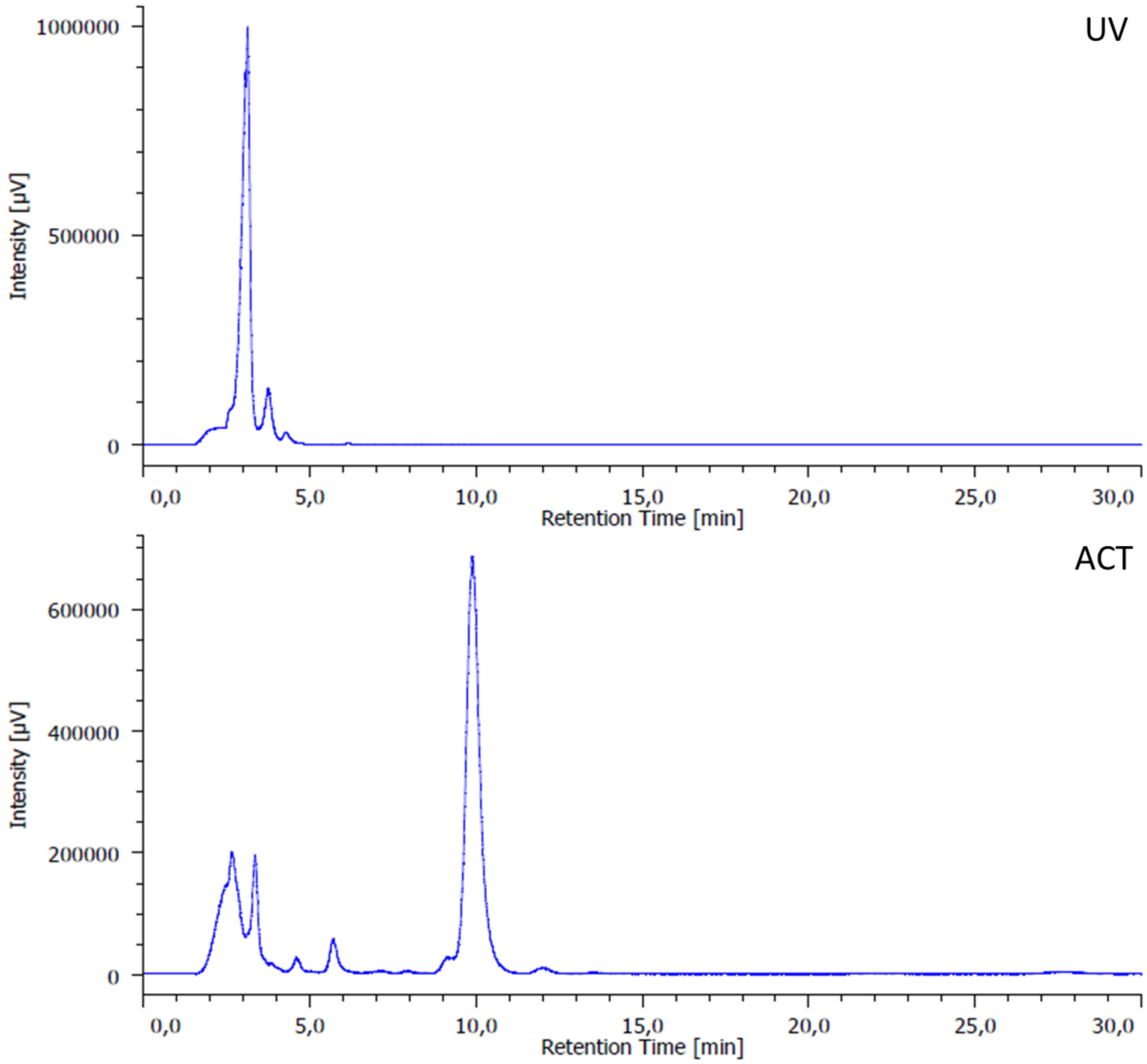

Preparative HPLC chromatogram of [^{11}C]2 (Reprospher C18-DE, MeCN/H_2O/DIPA 60:40:0.1, 3 mL·min^{-1}, 254 nm).

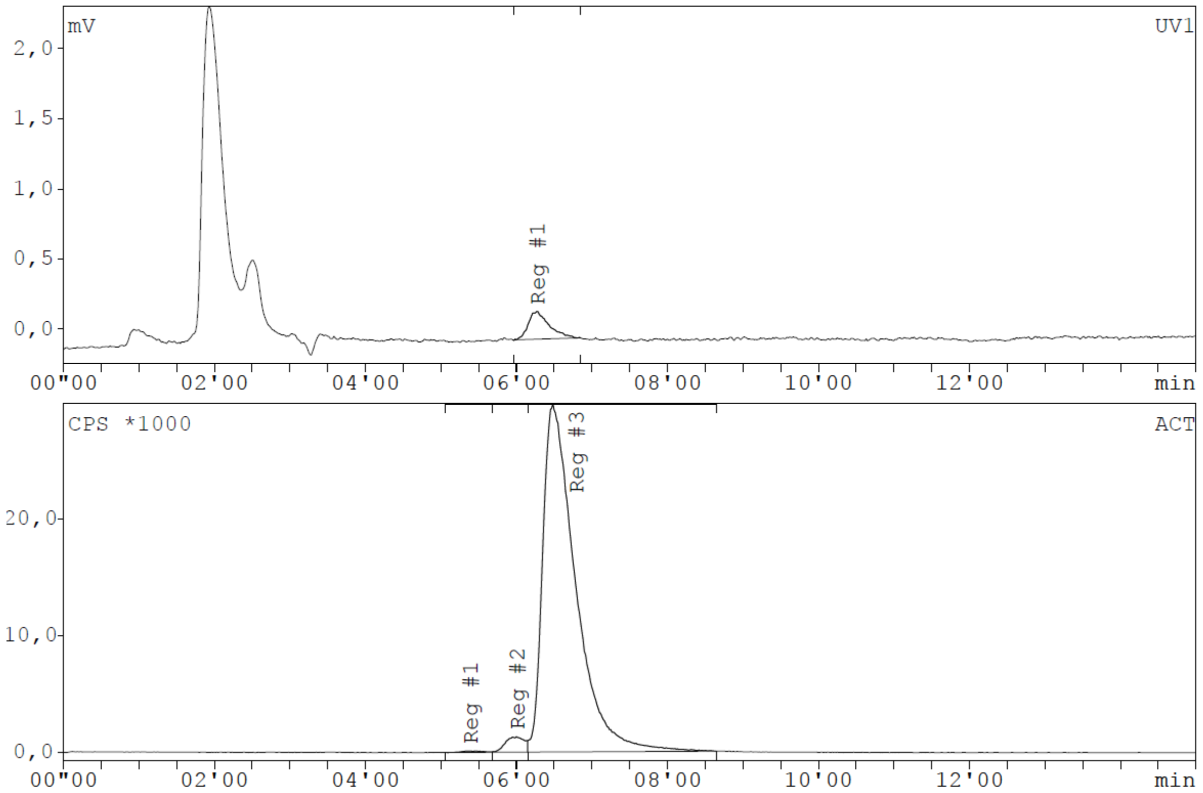

Analytical HPLC chromatogram of [^{11}C]2 formulation (Platinum C18, MeCN/H_2O/DIPA 50:50:0.1, 1 mL·min^{-1}, 285 nm).

2-chloro-*N*-((3,5-difluoroadamantan-1-yl)methyl)-5-[^{11}C]methoxybenzamide ([^{11}C]3)

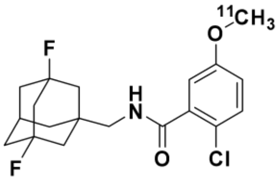

In a procedure analogous to that given above for [^{11}C]**1**, [^{11}C]**3** was obtained from precursor **7** (0.5 mg, 1.4 µmol). After preparative HPLC on a Reprospher C18-DE 5 µm (50 x 8 mm) column with MeCN/H_2O/DIPA (55:45:0.1, *v*/*v*/*v*) as eluent at a flow rate of 3 mL·min^{-1} (t_R = 11 min) and subsequent formulation identical to the procedure described for [^{11}C]**2**, [^{11}C]**3** was obtained in a RCY of 41 ± 9% (n = 3), calculated from [^{11}C]CO_2, with a radiochemical purity >99%, A_m of 221 ±119 GBq·µmol^{-1} (n = 3) at EOS and an overall synthesis time of 35-40 min. The identity of the product was confirmed by analytical HPLC using co-injection of the product and non-labelled **3** (Platinum C18 5 µm (4.6 x 250 mm) column using MeCN/H_2O/DIPA (50:50:0.1, *v*/*v*/*v*) as eluent at a flow rate of 1 mL·min^{-1}, t_R = 5.4 min).

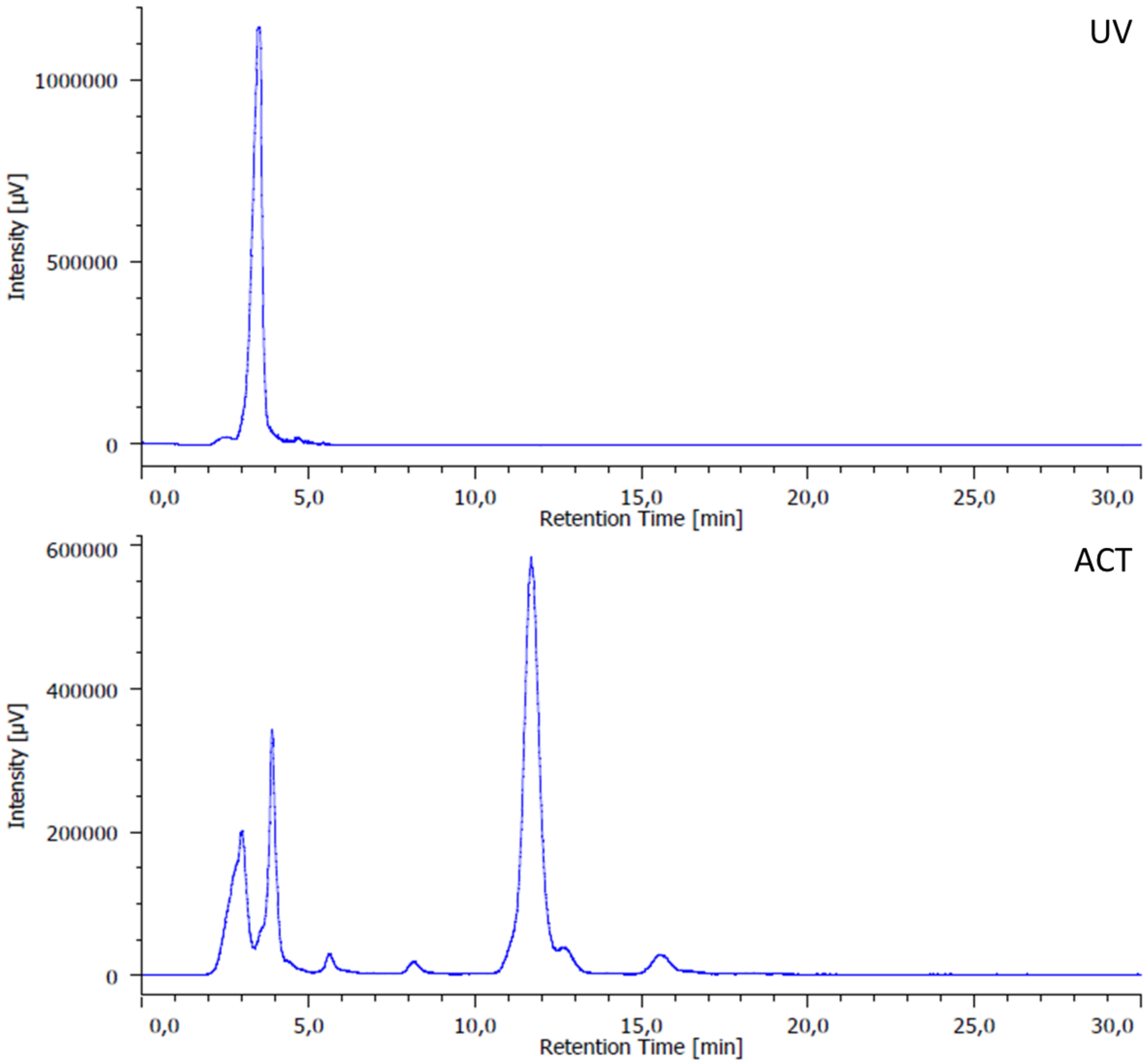

Preparative HPLC chromatogram of [^{11}C]3 (Reprospher C18-DE, MeCN/H_2O/DIPA 55:45:0.1, 3 mL·min^{-1}, 254 nm).

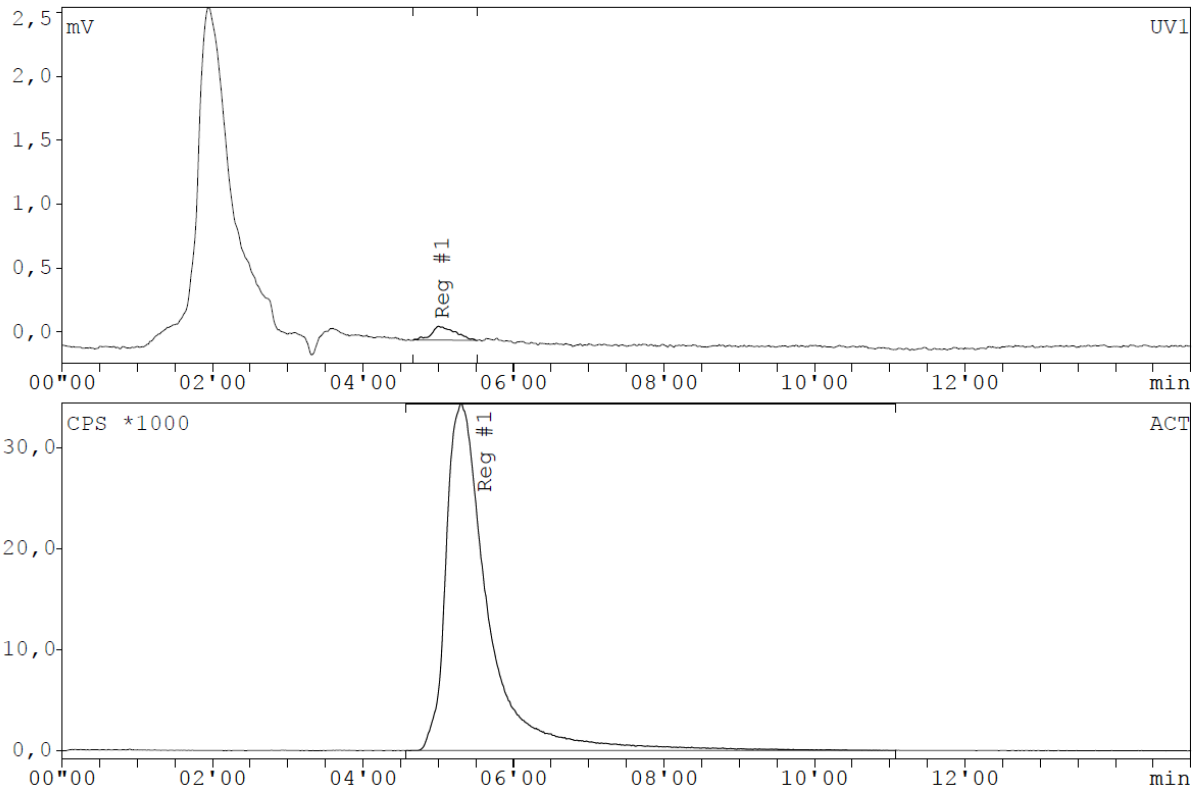

Analytical HPLC chromatogram of [^{11}C]3 formulation (Platinum C18, MeCN/H_2O/DIPA 50:50:0.1, 1 mL·min^{-1}, 285 nm).

2-chloro-5-[^{11}C]methoxy-*N*-((3,5,7-trifluoroadamantan-1-yl)methyl)benzamide ([^{11}C]SMW139)

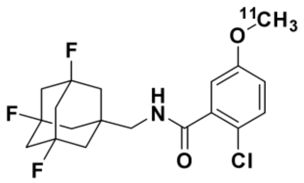

In a procedure analogous to that given above for [^{11}C]**1**, [^{11}C]SMW139 was obtained from precursor **8** (0.5 mg, 1.3 µmol). After preparative HPLC on an Alltima C18 5 µm (250x10 mm) column (Grace, Columbia MD, USA) using MeCN/H_2O/TFA 50:50:0.1, *v*/*v*/*v* at a flow rate of 5 mL·min^{-1} (t_R = 16 min) and subsequent formulation identical to the procedure described for [^{11}C]**2**, [^{11}C]SMW139 was obtained in a RCY of 37 ± 9% (n = 10), calculated from [^{11}C]CO_2, with a radiochemical purity >96%, A_m of 209 ± 83 GBq·µmol^{-1} (n = 10) at EOS and an overall synthesis time of 35-40 min. The identity of the product was confirmed by analytical HPLC using co-injection of the product and non-labelled SMW139 (Platinum C18 5 µm (4.6 x 250 mm) column using MeCN/H_2O/TFA (45:55:0.1, *v*/*v*/*v*) as eluent at a flow rate of 1 mL·min^{-1}, t_R = 12 min).

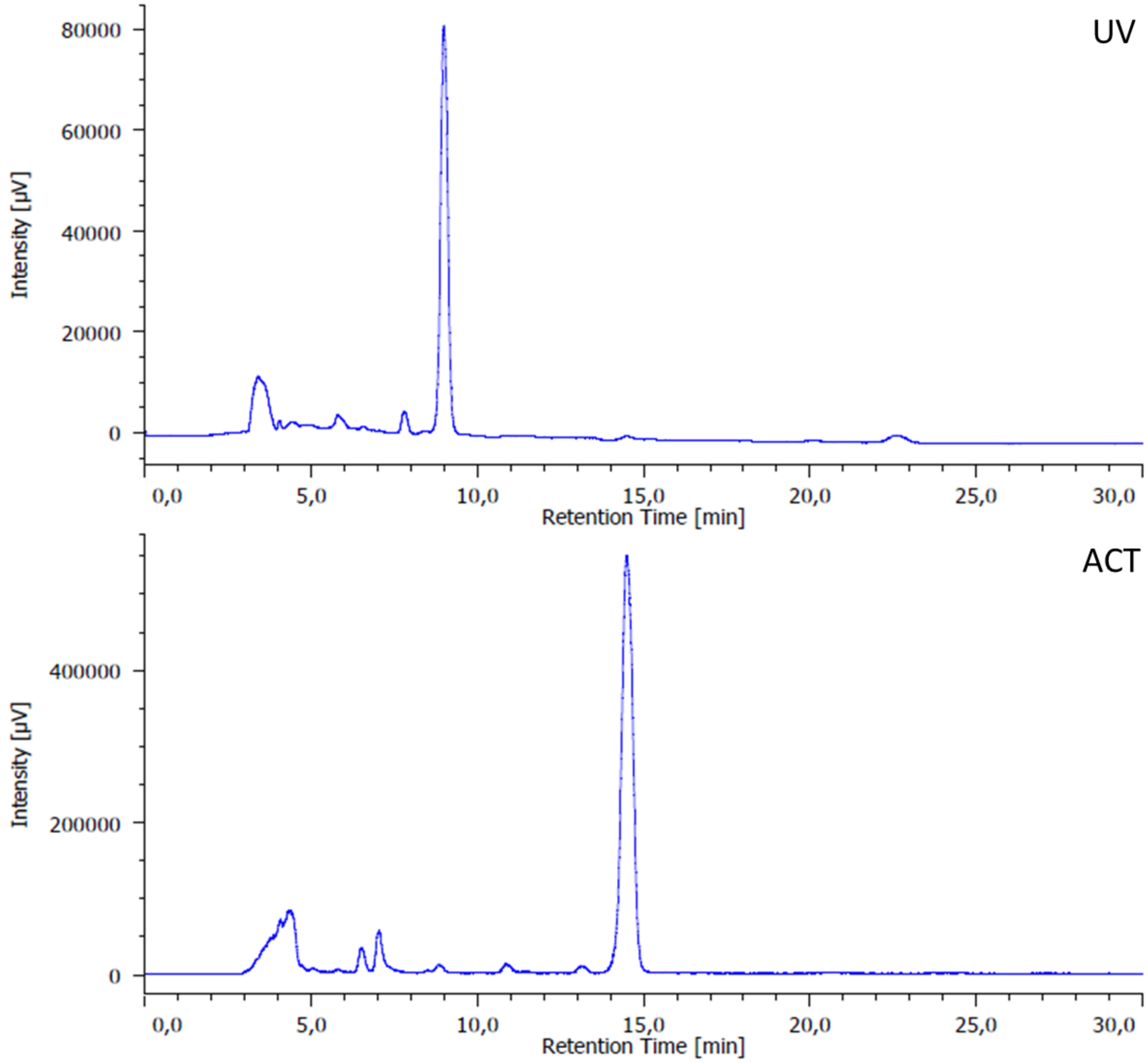

Preparative HPLC chromatogram of [^{11}C]SMW139 (Alltima C18, MeCN/H_2O/TFA 50:50:0.1, 5 mL·min^{-1}, 254 nm).

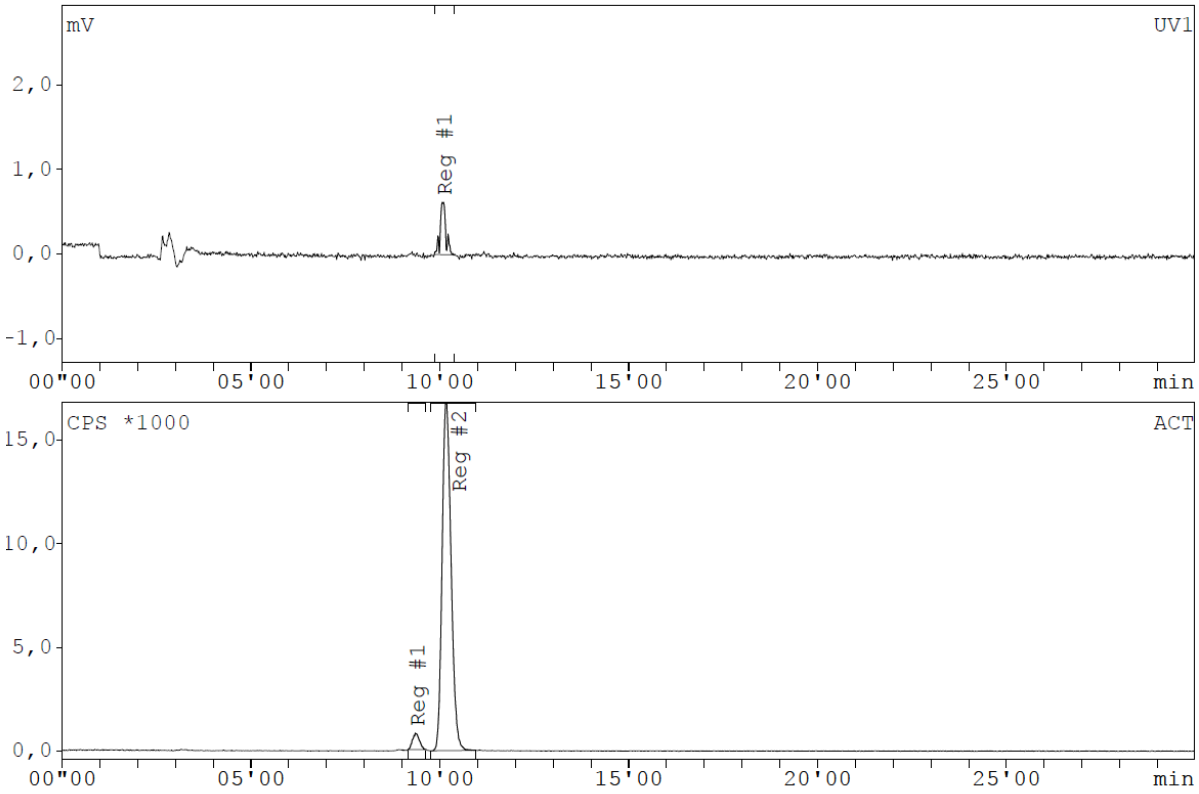

Analytical HPLC chromatogram of [^{11}C]SMW139 formulation (Platinum C18, MeCN/H_2O/TFA 45:55:0.1, 1 mL·min^{-1}, 285 nm).

Determination of LogD$_{oct,7.4}$

The distribution of radiolabelled compound between 1-octanol and 0.2 M phosphate buffer (pH = 7.4) was measured at room temperature. Briefly, 1 mL of a 20 MBq·mL^{-1} solution of radiolabelled compound in 0.2 M phosphate buffer (pH = 7.4) was mixed with 1 mL of 1-octanol for 1 min using a vortex mixer. After a settling period of 30 min, three samples of 100 µL were taken from both layers. All samples were counted for radioactivity in a Wallac Universal Gammacounter 1282 (PerkinElmer, Waltham, MA, USA). The LogD$_{oct,7.4}$ value was calculated according to LogD$_{oct,7.4}$ = ^{10}Log(A_{oct}/A_{buffer}), where A_{oct} and A_{buffer} represent average radioactivity counts of the three 1-octanol and the three buffer samples.

In vitro and *in vivo* evaluation

General

Healthy Wistar male and female rats were obtained from Harlan Netherlands B.V. (Horst, the Netherlands), Charles River ('s Hertogenbosch, the Netherlands or Tranent, Edinburgh, United Kingdom) and Janvier (France). All animal studies were conducted in accordance with the European Community Council Directive 2010/63/EU or UK Home Office regulations for laboratory animal ethics and welfare, and experiments were approved by the local animal ethics committees (Dierexperimentencommissie (DEC) of the VU and VUmc Amsterdam; KU Leuven University Ethics Committee for Animals; UK Home Office and Animal Welfare and Ethical Review Body at Pharmidex Ltd., London, UK).

Human brain tissue samples from Alzheimer's disease patients and non-neurological control cases were obtained from The Netherlands Brain Bank (NBB, www.brainbank.nl), Netherlands Institute for Neuroscience (Amsterdam, the Netherlands). The NBB's procedures are in accordance with all national laws and regulations and have been approved by the Medical Ethics Committee of the VU University Medical Center (Amsterdam, the Netherlands). All donors or their next of kin gave written informed consent for a brain autopsy and the use of the material and clinical information for research purposes. All tissue samples were handled according to Dutch national ethical guidelines (Code for Proper Secondary Use of Human Tissue, Dutch Federation of Medical Scientific Societies).

Ex vivo biodistribution

Healthy male Wistar rats (211-460 g) were injected with 13-23 MBq (at start of experiment) of formulated tracer in the tail vein under isoflurane anaesthesia (4% and 2% in 1 $L{\cdot}min^{-1}$ oxygen for induction and maintenance, respectively). At 5, 15, 30 and 45 min post injection (n = 3 per time point), rats were anaesthetised, blood was taken by heart puncture and subsequently animals were euthanized and dissected. Blood, heart, lungs, liver, kidneys, spleen, olfactory bulbs, hippocampus, striatum, cerebral cortex, cerebellum and the rest of the brain were collected, weighed and counted for radioactivity in a Wallac Universal Gammacounter 1282 (Turku, Finland). Biodistribution data were

expressed as standardised uptake value (SUV) to correct for differences in animal weight between experiments. SUVs were calculated using the following formula:

$$SUV = \frac{cpm_{organ}/cpm_{total}x100}{weight_{organ}(g)/bodyweight\ (g)}$$

Ex vivo metabolite analysis

Healthy male and female Wistar rats (254-440 g) were injected with 30-40 MBq (at start of experiment) of formulated tracer in the tail vein under isoflurane anaesthesia. Rats were sacrificed at 15 and 45 min (n = 3 per time point). Blood samples were obtained and the brain was excised. Blood samples were collected in heparin tubes (BD Vacutainer NH 119 I.U., 7 mL, BD Vacutainer Systems, Plymouth, UK) and centrifuged at 4,000 rpm for 5 min at 4 °C (Hettich Universal 32, Andreas Hettich GmbH & Co.KG, Tuttlingen, Germany). Plasma supernatant was separated from blood cells and loaded onto a tC18 Sep-Pak (Waters, Milford, MA, USA), followed by washing with 3 mL of water to obtain the polar fraction. The non-polar fraction was then eluted with 2 mL of MeOH and 1 mL of water and further analysed by HPLC. The brain was put in a falcon tube containing 4 mL MeCN/H_2O (50:50 *v/v*) and homogenised with a disperser (IKA T18 B Ultra-Turrax, IKA®-Werke GmbH & Co.KG, Staufen, Germany) before centrifugation (5 min, 4,000 rpm, 20 ºC, Hettich Universal 32). Supernatant was separated from brain precipitate and analysed by HPLC. Analytical HPLC was performed with Dionex (Sunnyvale, CA, USA) UltiMate 3000 HPLC equipment with Chromeleon software (version 6.8) on a Gemini C18 5-µm (10 x 250 mm) column (Phenomenex, Torrance, CA, USA) with gradient and a mixture of MeCN (A) and 0.1% DIPA in water (B) as eluent according to the following scheme: 0 min, 60% B at 0.25 mL min^{-1}; 0.5 min, 60% B at 4 mL·min^{-1}; 5.0 min, 10% B at 4 mL·min $^{-1}$; 12.0 min, 10% B at 4 mL·min^{-1}; 13.0 min, 60% B at 4.0 mL·min^{-1}; 14.5 min, 60% B at 4 mL·min^{-}1; and 15 min, 60% B at 0.25 mL·min^{-1}. All separate fractions were counted for radioactivity in a Wizard Gammacounter 1470 or 2480 (Wallac/PerkinElmer, Waltham, MA, USA).

Stereotactic injection of viral vector

Adeno-associated viral (AAV) vectors overexpressing *h*P2X$_7$ and *e*GFP were constructed and produced as described by Ory *et al*. [2]. Female Wistar rats (n=7) were anaesthetised by intraperitoneal (i.p.) injection of ketamine (75 mg·kg^{-1}); Ketalar, Pfizer, Brussels, Belgium) and medetomidin (1 mg·kg^{-1}; Domitor, Pfizer) and positioned in a stereotactic head frame (Stoelting, Wood Dale, IL, USA). Right and left striatum were injected with 4 µL of the *h*P2X$_7$R vector (rAAV_3flag-hP2X7R) and of the control vector (rAAV_3flag-eGFP), respectively, at a rate of 0.25 µL·min^{-1}, using a 30-gauge needle connected to a 10 µL Hamilton syringe. Coordinates used for striatal injection of the *h*P2X$_7$R vector were: anteroposterior 0 cm, lateral -0.28 cm and dorsoventral -0.64 cm relative to bregma. Coordinates used for striatal injection of the *e*GFP vector were: anteroposterior 0 cm, lateral +0.28 cm and dorsoventral -0.64 cm relative to bregma. After injection, the needle was kept in place for another 5 minutes before removing it slowly. To reverse the anaesthesia, the rats were injected intraperitoneally with 0.5 mg·kg^{-1} atipamezol (Antisedan, Orion Pharma, Newburry, Berkshire, UK). Scanning experiments were performed at 5 and 11 weeks after viral vector injection. Rats were sacrificed for *in vitro* autoradiography at 3 (n=2) and 12 (n=5) weeks after vector injection.

In vitro autoradiography

Snap frozen brains of female Wistar rats (rAAV_3flag-*h*P2X$_7$R in right striatum; rAAV_3flag-eGFP in left striatum; sacrificed 3 weeks (n = 2) or 12 weeks (n = 5) after vector injection) were cut on a Leica CM3050 S cryostat (Leica Biosystems, Nussloch, Germany) at -19 °C in transversal sections of 20 µm, thaw-mounted on SuperFrost™ Plus (Thermo Fisher Scientific, Waltham, MA, USA) object glasses, dried overnight on silica at 4 °C and then stored at -20 °C until use. Snap frozen human post-mortem brain tissue was cut on a Leica CM1850 cryostat at -15 °C in sections of 20 µm, thaw-mounted on SuperFrost™ Plus Gold (Thermo Fisher Scientific) object glasses and left in a storage box containing silica at room temperature for 1 h before drying overnight on silica at 4 °C, after which the sections were stored at -80 °C until use. Upon use, sections were thawed to room temperature for an hour before washing the sections in 5 mM Tris-HCl buffer (pH 7.4) at room temperature (3x 5 min). Sections were dried in a cold stream of air before incubation (30 min) with 5 mM Tris-HCl buffer (pH 7.4)

containing either [^{11}C]SMW139 (28 nM) alone or tracer and a blocking compound (A-740003 (synthesised in-house) or JNJ-47965567 (Tocris Bioscience, Bristol, United Kingdom) at 10 µM concentration. After incubation, sections were washed (3x 1.5 min) in ice-cold 5 mM Tris-HCl buffer (pH 7.4) followed by a dip in ice-cold demineralised water. Sections were dried in a stream of air and then exposed to a storage phosphor screen (BAS-IP SR 2040 E, GE Healthcare Europe GmbH, Eindhoven, the Netherlands) and the screen was read out on a Typhoon FLA 7000 phosphor imager (GE Healthcare Europe GmbH, Eindhoven, the Netherlands). After exposure, human post-mortem sections were stored at -80 °C for immunohistochemical staining (IHC). Images were analysed and quantified using ImageQuantTL Analysis Toolbox (version 8.1, GE Healthcare). In case of the rat brain sections, regions of interest (ROIs) were defined around the striatum. In case of human post-mortem brain material, ROIs were defined based on IHC. Results are expressed as mean intensity, or as percentage of tracer binding, relative to total tracer binding in the contralateral striatum. Experiments with rAAV_3flag-*h*P2X$_7$R injected rat brains were performed as a duplicate of quadruplicates. Experiments with human post mortem brain tissue were performed as duplicate (per patient). Statistical analysis of quantitative autoradiography data (unpaired t test, two-tailed) was performed with GraphPad Prism (Version 5.02, GraphPad Software Inc., La Jolla, CA, USA)

Immunohistochemical staining

For immunohistochemical analysis, frozen 5 µm thick sections were mounted on coated glass slides (Menzel Gläser Superfrost PLUS, Thermo Scientific). Sections were fixed by immersion in acetone for 10 min, followed by washing in phosphate buffered saline (PBS, pH 7.4). Between the subsequent incubation steps, sections were washed extensively with PBS. Sections were treated with 0.3% H_2O_2 in PBS for 30 min to block endogenous peroxidase. Mouse monoclonal anti-phospho-tau (AT8 for tau pSer202 and pThr205, 1:800, Pierce Biotechnology), mouse monoclonal anti-Aβ (IC16 1:200, Prof. C. Korth, Heinrich Heine University Düsseldorf, Germany), Rabbit polyclonal anti-Iba1 (1:3200, Wako), mouse monoclonal anti–MHC class II (CR3/43, 1:200, Dako), mouse monoclonal anti-CD68 (KP1, 1:600, Dako), and rabbit polyclonal anti-P2X7 (ab77413, 1:600, Abcam) were diluted in antibody diluent (Immunologic, Duiven, The Netherlands) and incubated overnight at room temperature. Omission of

the primary antibodies served as a negative control. Secondary EnVison^{TM} HRP goat anti-rabbit/mouse antibody (EV-GαM^{HRP}, Dako) incubation was for 30 min at room temperature. As chromogen 3,3-diaminobenzidine (Dako) was used and nuclei were counterstained with haematoxylin. Sections were dehydrated and mounted with coverslips using Quick-D mounting medium (BDH Laboratories Supplies, Poole, England). Immunoreactivity was determined blinded to the pathological and clinical diagnosis.

Slides were imaged with a Zeiss Axio Scan.Z1 slide scanner using a 20x objective. Six different areas representative of the whole section were extracted for quantification. The percentage of the area showing immunoreactivity for a specific antibody (area fraction) was determined using MacBiophotonics Image-J software (version 1.48). Student's t-test was used to determine differences between AD and control cases. Results are expressed as mean ± standard deviation (SD). A p-value of <0.05 was considered significant.

In vivo PET imaging

Dynamic PET imaging was performed using dedicated small animal NanoPET/CT and NanoPET/MR scanners (Mediso Ltd., Budapest, Hungary) [3,4] which are equipped with identical PET components. Female Wistar rats (n = 4; rAAV-3flag-hP2X$_7$R in right striatum; rAAV-3flag-eGFP in left striatum) were anaesthetised with 4% and 2% isoflurane in oxygen (1 L·min^{-1}) for induction and maintenance, respectively. Rats were positioned on the scanner bed and their respiratory rate was monitored during the entire scan, and anaesthesia was adjusted whenever required. Dynamic PET scans were acquired immediately after intravenous (i.v.) administration of 16-20 MBq of [^{11}C]SMW139 via the tail vein. For blocking experiments, rats were injected subcutaneously with JNJ-47965567 (30 mg·kg^{-1}) in 30% (2-hydroxypropyl)-β-cyclodextrin in water for injection 45 min prior to tracer injection. PET scans were acquired in list mode and rebinned into the following frame sequence: 4 × 5, 4 x 10, 2 x 30, 3x 60, 2 x 300, 1 x 600, 1 x 900 and 1 x 1200 s. Reconstruction was performed using a fully 3-dimensional reconstruction algorithm (Tera-Tomo^{TM}, Mediso Ltd.) with 4 iterations and 6 subsets, and an isotropic 0.4 mm voxel dimension. Images were analysed using the freely available AMIDE software (version 0.9.2; http://amide.sourceforge.net). 3D ellipsoid ROIs (dimensions x = 3.4 mm, y = 3.6 mm, z = 2.9

mm) were drawn within ipsi- and contralateral striata. Results are expressed as standardised uptake values (SUVs) and error bars indicate standard deviation. SUV were calculated as follows, with c_{img} in MBq/mL, injected dose in MBq and bodyweight in kg:

$$SUV(t) = \frac{C_{img}(t)}{injected\ dose/bodyweight}$$

An unpaired two-tailed t-test was used for statistical analysis of PET data, which was performed using GraphPad Prism (Version 5.02, GraphPad Software Inc., La Jolla, CA, USA). PET scans were performed at 5 (baseline) and 11 (pre-treatment with JNJ-47965567) weeks after vector injection, and rats were allowed to recover for 1 week after scanning. After 1 week of recovery following the final scan (12 weeks after stereotactic vector injection) animals were sacrificed, and brains were excised and stored at -80 °C until use.

Extended single microdose toxicity study

A single microdose toxicity study according to EMEA guideline EMA/CPMP/ICH/286/1995 (December 2009) was performed in healthy Wistar rats at a 1000 times higher dose than the maximal dose expected for a single tracer injection in humans (0.12 $\mu g \cdot kg^{-1}$, suggesting a maximum of 400 MBq at a minimum specific activity of 18.5 $GBq \cdot \mu mol^{-1}$). Animals were allowed to acclimatise for 10 days, group-housed and in an enriched environment with food and water available *ad libitum*, and with a 12-hour light-dark cycle in a temperature controlled room. Post-dosing, animals were single-housed, but with the ability to communicate. SMW139 was formulated in 6.7% EtOH in acetate/citrate buffer (citric acid monohydrate (8 $mg \cdot mL^{-1}$), sodium citrate dehydrate (16 $mg \cdot mL^{-1}$) and sodium acetate trihydrate (7 $mg \cdot mL^{-1}$); pH 5.2) with 2.5% Tween-80. Male and female Wistar rats (244-355 g; Charles River, Tranent, Edinburgh, United Kingdom) were injected intravenously via the tail vein under light isoflurane anaesthetics with formulated SMW139 (1 $mL \cdot kg^{-1}$, corresponding to a single dose of 0.12 $mg \cdot kg^{-1}$; female n = 10; male n = 10), or vehicle only (1 $mL \cdot kg^{-1}$; female n = 10; male n = 10) and animals were sacrificed 24 hours (SMW139; female n = 5; male n = 5; vehicle; female n = 5; male n = 5) or 14 days (SMW139; female n = 5; male n = 5; vehicle; female n = 5; male n = 5) post-dose by exsanguination

from the heart chamber under terminal isoflurane anaesthesia. Parameters assessed were clinical signs and mortality (0.5 h, 1 h, 2 h, 4h post-dose; followed by twice a day with a minimum of 4 h in between), changes in body weight (days 0, 1, 3, 6, 10, 13), food consumption (days 0-3, 3-6, 6-10, 10-13), terminal haematology, coagulation and clinical chemistry parameters, gross pathology and organ weights. For statistical analysis between groups, ANOVA followed by Tukey's pairwise comparisons test, was performed.

STUDY SUMMARY

The objective of this study was to determine the systemic acute toxicity of the INMiND compound SMW139 administered by intravenous injection in Wistar rats. This study was intended to provide information on major toxic effects and target organs.

The Test Item SMW139 was provided by the Sponsor and formulated according to Sponsor's instructions at the Testing Facility in the vehicle provided by the Sponsor, containing 6.7% ethanol in acetate/citrate buffer (pH 5.2) with 2.5% of Tween-80. Vehicle control combined with matrix 96% EtOH was preformulated by the Sponsor and was ready for use. Both test item and vehicle control have met the GLP requirements for non-clinical *in vivo* testing, as acknowledged by the sponsor, and a Certificate of Analysis was provided by the Sponsor.

The study consisted of two phases, a preliminary Sighting Phase designed to estimate the suitable non-toxic dose level followed by a Main Phase. The later was subdivided into Phases I and II to reduce the potential animal suffering in line with ethical scientific approach and British Home Office regulations.

Animals were dosed by intravenous bolus administration using the single dose of SMW139 (test article) or vehicle control formulation at a dose volume 1mL/kg. A preliminary Sighting study confirmed a safe dose level for SMW139 to be 0.12mg/kg, which was utilised for the Main Phase. Main Phase consisted of Group 1: 5 male and 5 female rats receiving vehicle control at 0.00mg/kg and 1mL/kg; Group 2: 5 male and 5 female rats receiving SMW139 at 0.12mg/kg and 1mL/kg; Group 3: 5 male and 5 female rats receiving vehicle control at 0.00mg/kg and 1mL/kg; Group 4: 5 male and 5 female rats receiving SMW139 at 0.12mg/kg and 1mL/kg. Main Phase I animals (Groups 1 and 2) were sacrificed at 24 hours post-dose, Main Phase II animals (Groups 3 and 4) were sacrificed at 14 days post-dose.

Parameters assessed were clinical signs, mortality, changes in body weight, food consumption, terminal haematology, coagulation and clinical chemistry parameters, gross pathology and organ weights.

Based on clinical observations, changes in body weight, food consumption, haematology, coagulation, clinical chemistry parameters, and necropsy findings, a single microdose administration of 0.12 mg/kg SMW139 by intravenous injection in Wistar rats showed no observed test item-related adverse effects of systemic acute toxicity throughout the experimental period of up to 14 days under the test conditions.

Supplementary figures

Ex vivo biodistribution

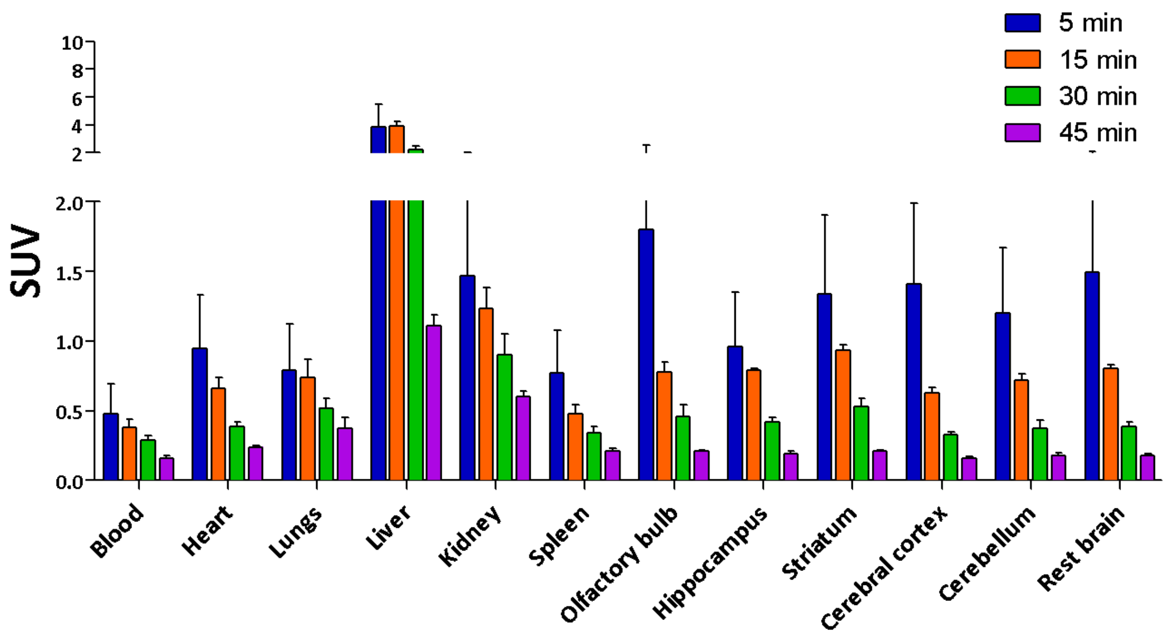

Figure S1: *Ex vivo* biodistribution following i.v. administration of [^{11}C]1 in healthy male Wistar rats (n=3 per tracer per time point). Data are expressed as standardised uptake value (SUV) ± standard error of the mean (SEM).

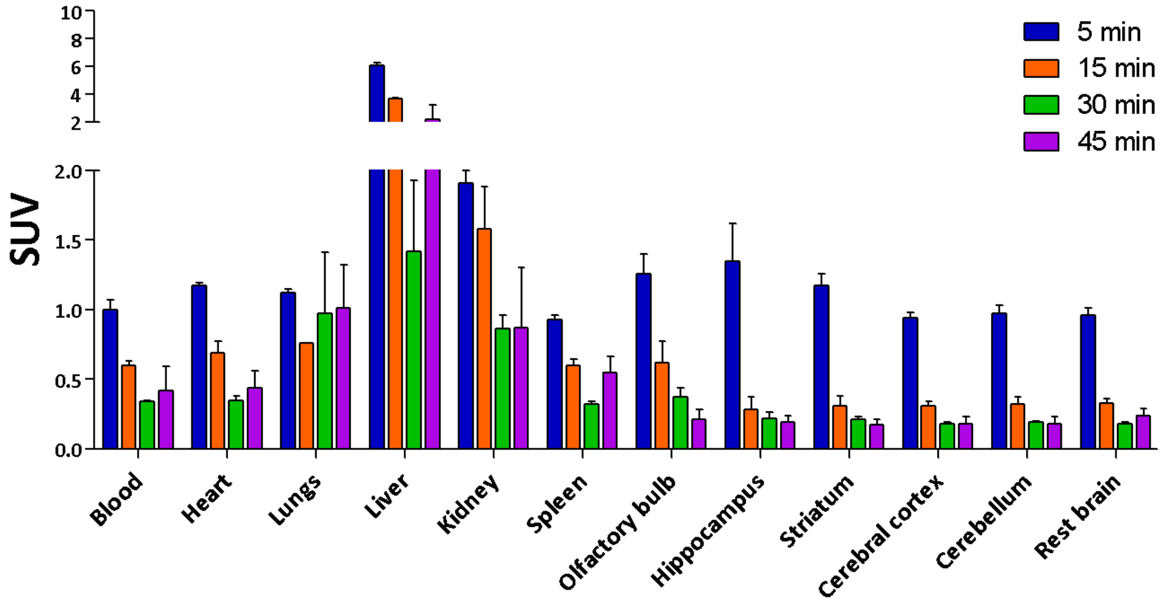

Figure S2: *Ex vivo* biodistribution following i.v. administration of [^{11}C]2 in healthy male Wistar rats (n=3 per tracer per time point). Data are expressed as SUV ± SEM.

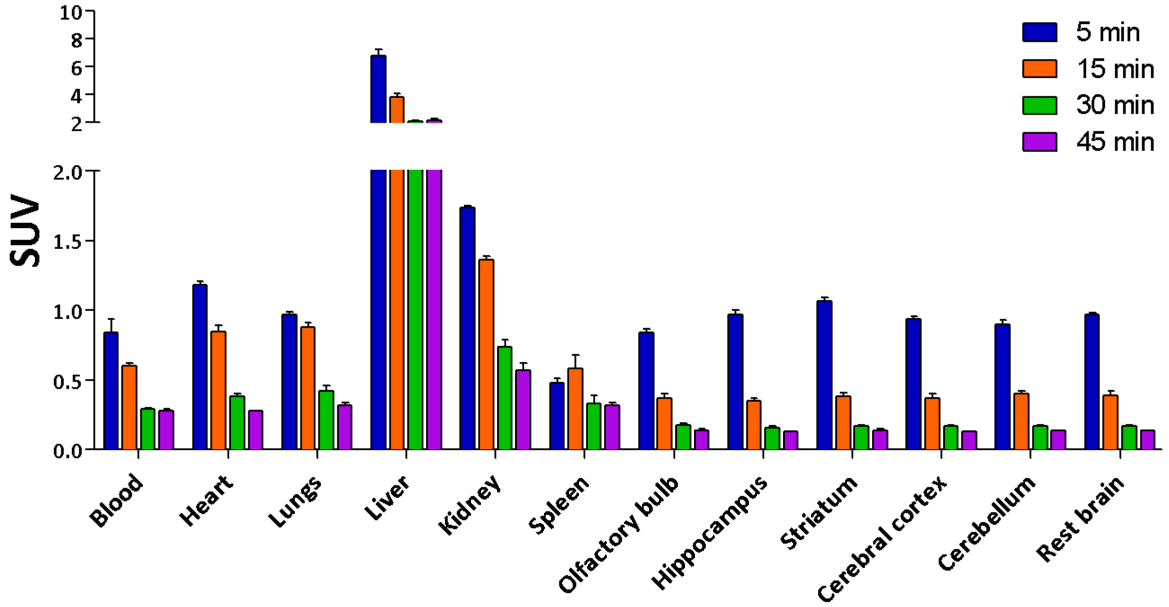

Figure S3: ***Ex vivo*** **biodistribution following i.v. administration of [^{11}C]3 in healthy male Wistar rats (n=3 per tracer per time point).** Data are expressed as SUV ± SEM.

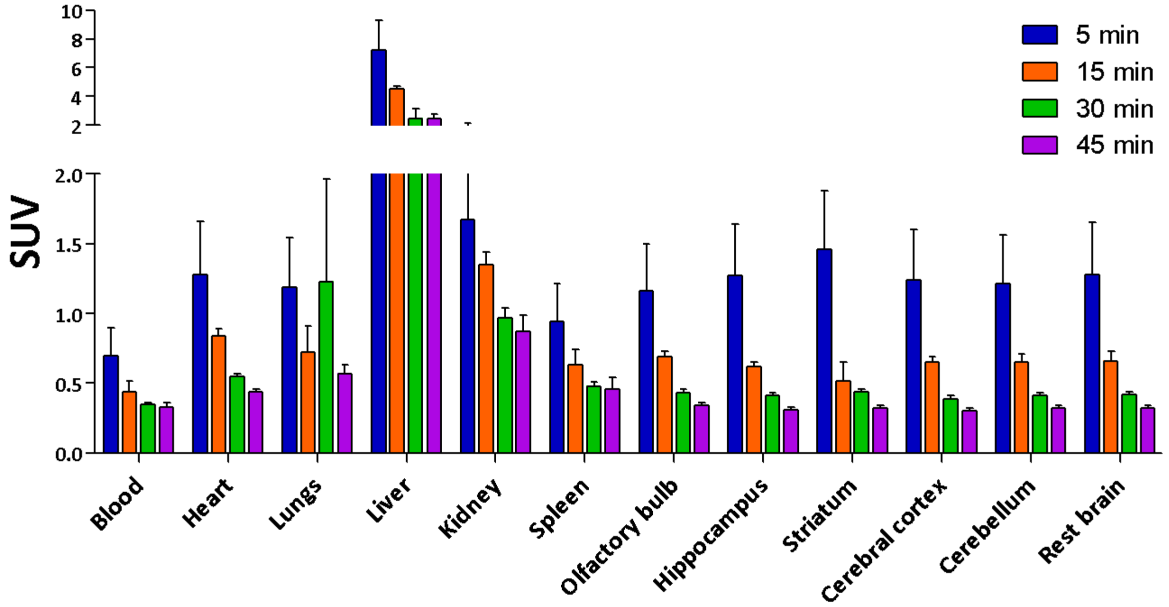

Figure S4: ***Ex vivo*** **biodistribution following i.v. administration of [^{11}C]SMW139 in healthy male Wistar rats (n=3 per tracer per time point).** Data are expressed as SUV ± SEM.

Metabolite analysis

Table S1: Metabolic profile of [^{11}C]SMW139 in female rAAV-*h*P2X$_7$R rats.

Plasma	Rat 1		Rat 2	
	**15 min**	**45 min**	**15 min**	**45 min**
**Intact tracer (%)**	78	50	63	32
**Non-polar metabolites (%)**	22	45	33	65
**Polar metabolites (%)**	0	4	5	3

PET imaging

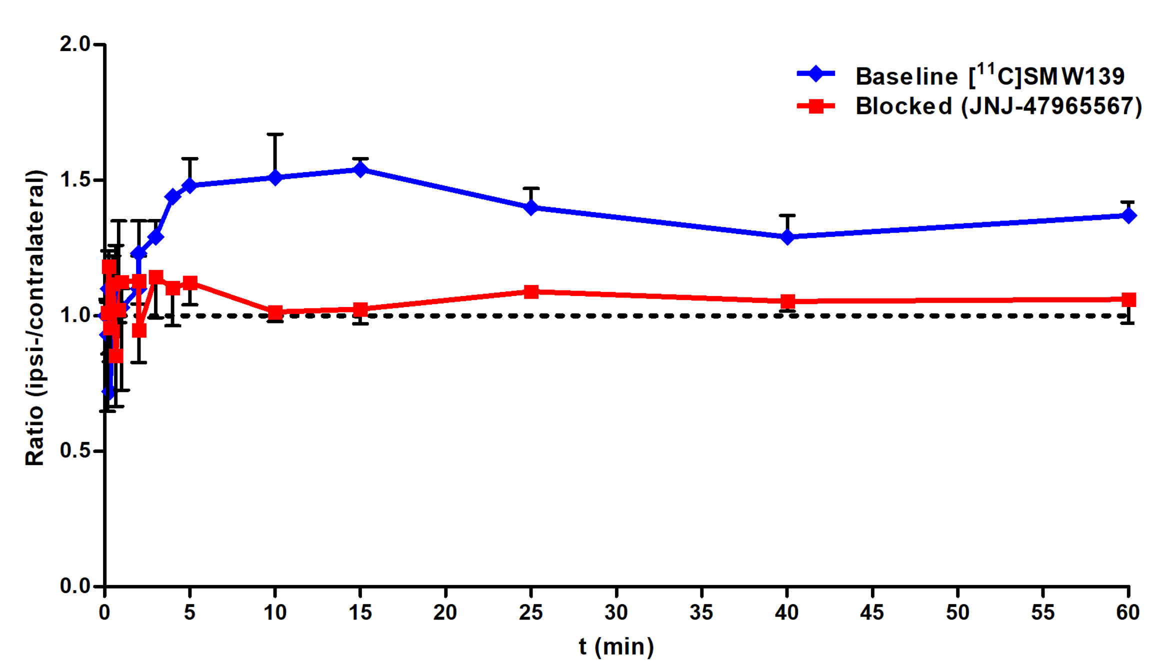

Figure S5: Ratio of [^{11}C]SMW139 binding in rAAV-*h*P2X$_7$R striatum over binding in contralateral striatum. Ratio is depicted in both baseline conditions (blue; diamonds) and blocking conditions (red; squares). Dotted line represents a ratio of 1.

References

[1] Armarego WLF, Chai CLL. Purification of Laboratory Materials. 5th ed. Elsevier Academic Press 2003.

[2] Ory D, Celen S, Gijsbers R, Van Den Haute C, Postnov A, Koole M, Vandeputte C, Andrés J-I, AlcazarJ, De Angelis M, Langlois X, Bhattacharya A, Schmidt M, Letavic MA, Vanduffel W, Van Laere K, Verbruggen A, Debyser Z, Bormans G. Preclinical evaluation of a P2X7 receptor selective radiotracer: positron emission tomography studies in a rat model with local overexpression of the human P2X7 receptor and in non-human primates. J. Nucl. Med. 2016;57:1436-1441.

[3] Szanda I, Mackewn J, Patay G, Major P, Sunassee K, Mullen GE, Nemeth G, Haemisch Y, Blower PJ, Marsden PK. National Electrical Manufacturers Association NU-4 Performance Evaluation of the PET Component of the NanoPET/CT Preclinical PET/CT Scanner. J. Nucl. Med. 2011;52;1741-1747.

[4] Nagy K, Tóth M, Major P, Patay G, Egri G, Häggkvist J, Varrone A, Farde L, Halldin C, Gulyás B. Performance Evaluation of the Small-Animal nanoScan PET/MRI System. J. Nucl. Med. 2013;54;1825-1832.
